# New Insights into Alzheimer’s Disease: Novel Pathogenesis, Drug Target and Delivery

**DOI:** 10.3390/pharmaceutics15041133

**Published:** 2023-04-03

**Authors:** Haishu Chen, Jinan Xu, Hanyuan Xu, Tiancheng Luo, Yihao Li, Ke Jiang, Yangping Shentu, Zhiqian Tong

**Affiliations:** 1 Oujiang Laboratory (Zhejiang Lab for Regenerative Medicine, Vision and Brain Health), Institute of Aging, Key Laboratory of Alzheimer’s Disease of Zhejiang Province, Zhejiang Provincial Clinical Research Center for Mental Disorders, The Affiliated Wenzhou Kangning Hospital, School of Mental Health, Wenzhou Medical University, Wenzhou 325035, China; 2 Cixi Biomedical Research Institute, Wenzhou Medical University, Wenzhou 325035, China; 3 Institute of Albert, Wenzhou Medical University, Wenzhou 325035, China; 4 Department of Pathology, The First Affiliated Hospital of Wenzhou Medical University, Wenzhou 325035, China

**Keywords:** Alzheimer’s disease, drug delivery, extracellular space, blood–brain barrier, formaldehyde, interstitial fluid

## Abstract

Alzheimer’s disease (AD), the most common type of dementia, is characterized by senile plaques composed of amyloid β protein (Aβ) and neurofilament tangles derived from the hyperphosphorylation of tau protein. However, the developed medicines targeting Aβ and tau have not obtained ideal clinical efficacy, which raises a challenge to the hypothesis that AD is Aβ cascade-induced. A critical problem of AD pathogenesis is which endogenous factor induces Aβ aggregation and tau phosphorylation. Recently, age-associated endogenous formaldehyde has been suggested to be a direct trigger for Aβ- and tau-related pathology. Another key issue is whether or not AD drugs are successfully delivered to the damaged neurons. Both the blood–brain barrier (BBB) and extracellular space (ECS) are the barriers for drug delivery. Unexpectedly, Aβ-related SP deposition in ECS slows down or stops interstitial fluid drainage in AD, which is the direct reason for drug delivery failure. Here, we propose a new pathogenesis and perspectives on the direction of AD drug development and drug delivery: (1) aging-related formaldehyde is a direct trigger for Aβ assembly and tau hyperphosphorylation, and the new target for AD therapy is formaldehyde; (2) nano-packaging and physical therapy may be the promising strategy for increasing BBB permeability and accelerating interstitial fluid drainage.

## 1. Introduction

Alzheimer’s disease (AD) is the most common neurodegenerative disease associated with progressive cognitive decline. AD is the major type of dementia in 60–70% of cases. By 2050, the number of new cases of dementia will be over 1 million per year worldwide [1]. Even though more than 600 billion dollars have been invested in drug developments for AD treatment, the outcomes are insufficient [2]. The failures of research and drug development for AD force us to reflect on two critical questions: (1) Which endogenous factor initiates AD occurrence? (2) Do AD drugs successfully reach the damaged neurons?

The hypothesized causes of AD include amyloid cascade, presenilin, tau hyperphosphorylation, cholinergic, calcium imbalance, oxidative stress, etc. Two main factors have been proposed as the critical triggers of AD: amyloid and tau [3]. These hypotheses suggest that eliminating amyloid and hyperphosphorylated tau could improve cognition in AD patients, while major drugs targeting amyloid and tau proteins could not improve cognitive functions in clinical trials. Recently, calcium imbalance and oxidative stress were found to play an important role in the development of AD [4,5].

**Figure 1:**
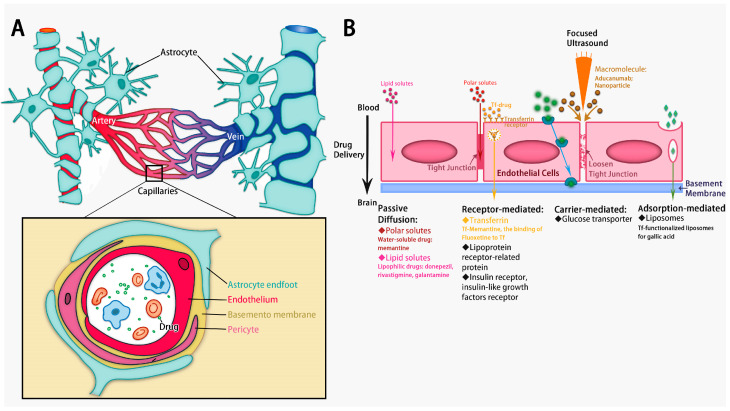
(**A**) The structure of the blood–brain barrier. (**B**) Alzheimer’s drug delivery to the BBB (Several ways to increase the AD drug delivery cross BBB. Focused ultrasound increases delivery of aducanumab [9]. Transferrin (Tf) increases delivery of memantine [10] and fluoxetine [11]. Tf-functionalized liposomes increase brain delivery of gallic acid [12]).

In fact, whether the medicines arrived at the targeted neurons in the brains is determined by at least two key structures, the blood–brain barrier (BBB) and brain extracellular space (ECS) [2]. The BBB is a barrier formed by vascular endothelial cells and a variety of glial cells. The BBB separates brain tissue from peripheral circulation and plays a major role in maintaining the brain’s microenvironments by preventing the entry of exogenous harmful substances into the brain [6] (Figure 1). Although the BBB is a great hurdle for drug delivery into the brain [7], the integrity of the BBB has been found to be impaired in AD [8]. Unexpectedly, the developed medicines for AD have still not met the clinical expectations; hence, the BBB may be not the primary reason for drug treatment failure.

**Figure 2:**
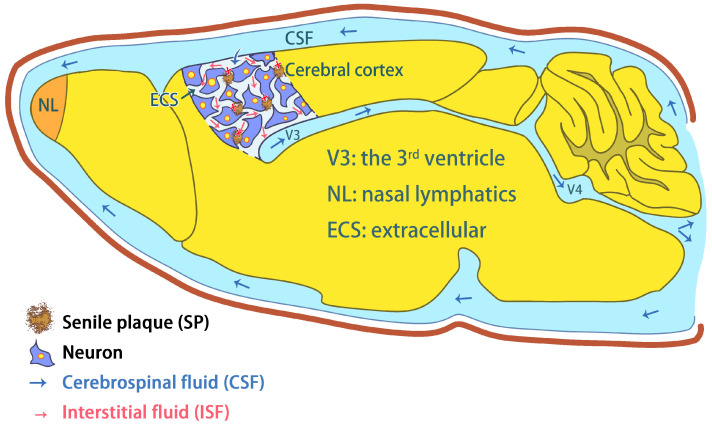
Interstitial fluid (ISF) drainage flows from the superficial cortex and then deep into the brain to the 3rd ventricle pouring into the cerebrospinal fluid (CSF). Finally, CSF flows into nasal lymphatics (NL) where the substances are exchanged with blood [18]. Formaldehyde-induced Aβ deposition in the ECS blocks the exchange between ISF and CSF.

The brain extracellular space (ECS) is a narrow gap between brain cells and neighboring cells, which account for approximately 20% of brain volume [13]. The ECS consists of interstitial fluid (ISF), the extracellular matrix (ECM) and other secretory molecules. Neurons and glial cells exchange substances and information in the ECS. The width of the ECS is only 38–64 nm; thus, medicines need to be smaller than this width in diameter in order to cross through the ECS [14]. The main function of ISF is to remove metabolic waste, provide nutrients, and act as a crucial medium for drug delivery [15]. Owing to the separation of dense myelinated fiber bundles, interstitial fluid drainage in the normal brain is regional. It may be difficult for medicines to reach effective concentrations in certain brain regions [16]. Studies have shown that formaldehyde induces Aβ misfolding and oligomerization [17] and senile plaques in extracellular space [18]. Notably, Aβ-related SP deposition in the extracellular space has been proven to slow down or stop ISF drainage in AD, thus actually blocking delivery into the brain [18,19] (Figure 2).

## 2. Developed Drugs Targeting Aβ and Tau for AD Therapy

Despite the huge investments in drug development, currently, only seven drugs for AD treatment have been approved for marketing by the Food and Drug Administration (FDA) [20,21]. These drugs fall into three categories: cholinesterase inhibitors, NMDA receptor antagonists, and antiβ amyloid (Aβ) monoclonal antibodies. Five of these drugs are used for symptomatic treatment, including donepezil, rivastigmine, galantamine, memantine, and a combination of memantine and donepezil. Recently, the remaining two drugs, Aducanumab and Lecanemab, were designed to be applied in the etiologic treatment of AD [22,23]. Disappointingly, compounds, peptides, and antibodies developed to target Aβ production, aggregation, and elimination as well as tau phosphorylation and aggregation have not met clinical expectations [24].

### 2.1. Drugs Targeting Aβ

Recently, antiamyloid therapies are a key research focus in terms of the therapeutic principle related to the Aβ cascade hypothesis. This hypothesis suggests that the deposition of Aβ in the brain is the main reason for AD occurrence. The 37–43 amino acid of Aβ is produced by its precursor, the β-amyloid precursor protein (APP). Three protease activities, those of α-, β-, and γ-secretase, are involved in Aβ generation [25]. The nontoxic Aβ monomer has several physiological functions [26]. For example, it is used in APP knockout mice to reduce Aβ-induced weight loss, abnormal muscle and neuronal development, and the number of neurotransmitters [27]. However, Aβ_42_ overloads are associated with the imbalance between Aβ production and clearance [28]. Aβ_42_ aggregation contributes to the formation of soluble Aβ oligomers (AβO) and insoluble Aβ protofibrils, the principal component of senile plaques (SPs) [29]. Toxic soluble Aβ oligomers induce neuroinflammation, cause dendritic spines of neuronal injury and inhibit long-term potentiation [30,31]. Remarkably, Aβ plaques cause neuronal death and memory impairment by blocking the ECS and ISF flow [18]. Aβ deposition also induces cerebral amyloid angiopathy and further exacerbates cognitive impairments [32,33]. In addition, Aβ induces tau phosphorylation through the PAX6 signaling pathways [34]. At present, the most developed drugs for AD treatment are antiamyloid drugs. For example, Aducanumab and Lecanemab have been approved for clinical treatment for AD patients [22,23].

#### 2.1.1. Drugs Used to Reduce Aβ Production

According to the different sites of APP digestion, the drugs used to reduce Aβ production fall in three categories: β-secretase inhibitors, γ-secretase inhibitors and modulators, and α-secretase activators.

β-secretase (BACE1) is one of the key enzymes for Aβ production. However, the development of β-secretase inhibitors often does not meet clinical expectations (Table 1). *Verubecestat*, a BACE1 inhibitor, was once considered a promising AD drug as it was found to reduce Aβ levels in the cerebrospinal fluid of rats, monkeys and AD patients in a preliminary study [35]. However, two Phase III clinical studies (NCT01739348 and NCT01953601) were terminated due to their inability to achieve the expected results. These AD patients experienced multiple treatment-related adverse effects, including rashes, falls and injuries, sleep disturbances, suicidal ideation, weight loss, and hair discoloration [36,37]. In addition, studies showed that Verubecestat may lead to accelerated volume reduction in the hippocampus (and other brain regions) in AD patients [38]. β-secretase inhibitors, such as *Lanabecestat*, *Elenbecestat*, *Umibecestat*, and *Atabecestat*, were discontinued due to a lack of clinical efficacy. Although these drugs inhibited BACE1 successfully, they did not improve cognitive functions in AD patient because BACE1 cleaves substrates for various functions, especially in neuron cell excitability regulation and axonal myelination [39]. Hence, the inhibition of BACE1 leads to many adverse effects and even exacerbates cognitive function deterioration.

γ-secretase inhibitors (GSIs) face a similar dilemma to that faced by β-secretase inhibitors (Table 1). Poor effectiveness and harmful side effects are the main reasons why these medications have been discontinued. For example, the γ-secretase enzyme cleaves more than 140 substrates, including APLP1 and APLP2, Notch, ErbB4, and p75 [40]. Reducing the hydrolysis of these substrates leads to a variety of adverse reactions. For example, *Semagacestat* leads to a deterioration in cognitive function, skin cancer, an increased risk of infection, and gastrointestinal bleeding by reducing Notch signaling [41]. *Avagacestat*, another γ-secretase inhibitor, also causes multiple adverse reactions, including nonmelanoma skin cancer and gastrointestinal symptoms [42]. As γ-secretase inhibitors increase the risk of cancer and cause cognitive decline, they may be an inappropriate target for the clinical treatment of AD [43]. The γ-secretase modulator (GSM) can selectively inhibit Aβ_42_ production without affecting the total amount of Aβ produced and Notch signaling. *Tarenflurbil*, a GSM, showed success in Phase II RCTs [44]. However, further RCTs on it ended due to its lack of efficacy and adverse effects including dizziness, anemia and infection after treatment [45].

α-secretase inducers increase APP hydrolysis to produce nonamyloid proteins. sAPPα, the cleavage fragments of APP, are involved in neurotrophic and neuroprotective functions [46]. Drugs may act through different signaling cascades to activate ADAM10 (α-secretase A Disintegrin and Metalloprotease 10, and α-secretase in neurons) and stimulate the cleavage of nonamyloid proteins [47]. Disulfiram induces the expression of ADAM10, reduces levels of Aβ plaques in the dentate gyri of AD mice and improves behavioral deficits [48]. Bryostatin1, a macrolide antitumor agent, reduces Aβ40 and Aβ42 in AD mice brain effectively and increases sAPPα secretion in AD patients [49]. Two Phase II RCTs of it finished. Cognitive enhancement was observed in advancing AD patients not receiving Memantine [50]. Acitretin, a synthetic retinoid, stimulates ADAM10 promoter activity and increases CSF sAPPα levels in patients with mild to moderate AD [51]. Epigallocatechin gallate (EGCG), a natural polyphenolic flavonoid, increases α-secretase cleavage activity and improves the cognitive function of APP/PS1 mice [52,53,54]. Additionally, EGCG leads to a reduction in neuroinflammation [52] and plays a crucial role in neuroprotection and prevention of Alzheimer’s disease [55]. A Phase 2/3 clinical trial of EGCG was recently completed, but no results have been published. There are a variety of natural compounds, including Cryptotanshinone [56,57], Ligustilide [58], Bilobalide [59,60] and Curcumin [61,62], that facilitate the activation of ADAM10 with potential neuroprotection in vivo.

**Table 1:** Alzheimer drugs targeting β-secretase, γ-secretase and α-secretase.

Drug Name	Drug Target	Phase	Effect in Clinical Trials	Status	Refs.
Umibecestat (CNP520)	β-secretase	Phase 2/3 (NCT03131453; NCT02565511)	Cognitive function decreased slightly, brain atrophy increased, weight loss	Discontinued	[63,64]
CTS-21166	β-secretase	Phase 1 (NCT00621010)	Reduced Aβ in plasma with long-lasting and well-tolerated effects	Discontinued	[65,66]
LY2811376	β-secretase	Phase 1 (NCT00838084)	Reduced Aβ in CSF *; adverse effects: retinal toxicity	Discontinued	[67]
LY2886721	β-secretase	Phase 1 Phase 2 (NCT01561430)	Reduced Aβ in plasma and CSF *; adverse effects: abnormal elevation of liver enzymes	Discontinued	[68]
AZD3839	β-secretase	Phase 1 (NCT01348737)	Slightly reduced Aβ in plasma at doses that did not disrupt cardiac activity	Completed	[69]
Verubecestat (MK-8931)	β-secretase	Phase 3 (NCT01953601)	Well-tolerated; reduced Aβ_40_ levels in CSF *	Discontinued	[70]
Lanabecestat	β-secretase	Phase 3 (NCT02972658; NCT02783573)	Reduced Aβ_40_ and Aβ_42_ levels in plasma and CSF *	Discontinued	[71]
Elenbecestat (E2609)	β-secretase	Phase 3 (NCT02956486)	Well-tolerated; reduced Aβ levels in plasma and CSF*; reduced BACE1 enzyme activity in CSF *; did not alter BACE1 levels	Discontinued	[72,73]
Atabecestat (JNJ-54861911)	β-secretase	Phase 2 Phase 3 (NCT02569398)	Reduced Aβ levels in CSF * and plasma; adverse effects: cognitive deterioration, and elevated liver enzymes	Discontinued	[74,75,76,77,78]
LY3202626	β-secretase	Phase 2 (NCT02791191; NCT03367403)	Resulted in high blood–brain barrier permeability; reduced Aβ_1-42_ in CSF *; no reduction in cognitive impairment and tau load	Discontinued	[79,80]
Semagacestat (LY450139)	γ-secretase	Phase 3 (NCT01035138; NCT00762411; NCT00594568)	Reduced the production of Aβ in patients; no reduction in cognitive impairment; adverse reactions: increased risk of skin cancer and infection	Discontinued	[41,81]
Avagacestat (BMS-708,163)	γ-secretase	Phase 2 (NCT00890890; NCT00810147)	Slightly reduced Aβ levels in CSF *; adverse reactions: gastrointestinal symptoms, skin diseases, and non-melanoma skin cancer	Discontinued	[82,83]
Tarenflurbil (R-flurbiprofen)	γ-secretase	Phase 3 (NCT00380276; NCT00380276; NCT00105547)	No reduction in cognitive impairment; adverse effects: dizziness, anemia and infection	Discontinued	[44,45]
PF-06648671 (Pfizer)	γ-secretase	Phase 1 (NCT02407353; NCT02440100)	Well-tolerated in healthy subjects; reduced plasma Aβ_40_ and Aβ_42_ and increased Aβ_37_ and Aβ_38_	Discontinued	[84]
CHF5074	γ-secretase	Phase 2 (NCT01303744)	Reduced inflammatory factor CD40 and TNF-α concentrations in CSF; improved executive function in ApoE4 gene carriers	Inactive	[85]
Bryostatin1	α-secretase	Phase 2 (NCT02431468; NCT04538066)	Reduced Aβ40 and Aβ42 and cognitive impairment	Active, not recruiting	[50]
Isotretinoin	α-secretase	Phase 1 Phase 2 (NCT01560585)	Adverse events in 2/3 participants	Terminated	[47]
EHT0202	α-secretase	Phase 2 (NCT00880412)	No significant effect	Completed	[86]
Acitretin	α-secretase	Phase 2 (NCT01078168)	Significantly increased CSF * APPs-α; safe and well-tolerated	Completed	[51]
Curcumin	α-secretase	Phase 2 (NCT00164749; NCT00099710; NCT01811381)	No effects on cognitive function and CFS * and plasma Aβ levels	Unknown	[47]

* Abbreviations: CSF, cerebrospinal fluid.

#### 2.1.2. Drugs Used to Prevent Aβ Aggregation

Since non-toxic Aβ monomers aggregate to form neurotoxic AβO and SPs [87], some drugs have been established to prevent Aβ aggregation (Table 2). *Tramiprosate* is an orally active natural amino acid with good BBB permeability. By stabilizing the multiligand encapsulation of the Aβ_42_ monomer, it inhibits Aβ oligomers and SP formation in AD mice [88]. Although it did not improve cognition in a treatment group, the results of magnetic resonance imaging (MRI) revealed that it reduces hippocampus atrophy in patients [89]. *Tramiprosate* and its precursor, *ALZ-801*, share a common metabolite, 3-sulfoniopropionic acid (3-SPA), which is associated with anti-Aβ oligomeric activity, good oral bioavailability and brain permeability [90]. *ALZ-801* produces positive biomarker results and improves cognitive functions in AD patients. ALZ-801 treatment is still being carried out in a Phase III RCT currently. The peptide sequence *KLVFF* retards Aβ aggregation and partially dissolves the Aβ oligomer [91]. Some studies have shown that several natural compounds could act as aggregation inhibitors [92], such as Brazilin [93], gx-50 [94], Curcumin [95], Epigallocatechin gallate [96,97], and Ginnalin A [98].

#### 2.1.3. Drugs Used to Promote Aβ Clearance

Another treatment strategy for AD patient is Aβ clearance through active and passive immunization (Table 3). After the administration of Aβ_42_ to PDAPP transgenic mice, SP formation was reduced through active immunization [104]. In contrast, passive immunization is achieved through Aβ antibody injection that directly reduces Aβ oligomers and senile plaques (SP). Although Aβ antibodies exhibit SP clearance, they also trigger a local inflammatory response and enhance vascular permeability [105]. Varying degrees of amyloid-related imaging abnormalities (ARIA) including cerebral edema (ARIA-E) and cerebral hemorrhage (ARIA-H) are developed after Aβ antibody therapy, which may aggravate cognitive impairments in AD patients.

*Aducanumab* is a fully human IgG1 monoclonal antibody with high affinity for the Aβ conformational epitope. It received accelerated approval from the FDA in June 2021 [124]. In transgenic mouse models of AD, Aducanumab enters into the brain and reduces soluble and insoluble Aβ levels. It also decreases brain Aβ deposition in patients with prodromal or mild AD [106]. Previous studies showed that it produced the most favorable effects among all Aβ monoclonal antibodies according to Phase III RCT results [107]. However, 41.3% of trial participants experienced ARIA during the trial period. A total of 1% to 2% of patients required hospitalization or experienced long-term side effects [108].

*Lecanemab* is a human IgG1 monoclonal antibody targeting protofibrils of soluble Aβ aggregates. In January 2023, the FDA granted accelerated approval for it. Preclinical trials have shown that it may protect neurons and decrease the amount of Aβ protofibrils in CSF [125,126]. In treatment groups, the treatment group has a 29.7% slower decline in Alzheimer’s disease composite scores (ADCOMS) at 18 months compared to the placebo group. However, approximately 12.6% developed cerebral edema and 26.4% of participants experienced infusion-related reactions [109,110]. A recent Phase III clinical trial showed that 18 months of treatment with it slowed the CDR-SB decline rate by approximately 27% and lessened the accumulation of tangles in the medial temporal lobe. Two-thirds of the treatment group became PET-Aβ-negative at 18 months [20].

Although these two Aβ antibodies have been approved, the developed drugs for the Aβ antibody encounter failure. The first generation antiAβ antibodies *Bapineuzumab* and *Solanezumab* did not improve clinical outcomes in mild to moderate AD. RCTs of *Crenezumab* and *Gantenerumab* ended for similar reasons. *Donanemab* is a humanized IgG1 monoclonal antibody that recognizes Aβ (p3-42), the N-terminal pyroglutamate of Aβ in amyloid plaques [117]. It decreases SPs rapidly and continuously in phase 1b [118]. In a Phase II RCT, it showed improvement in cognitive performances and daily activities in early AD patients [119].

### 2.2. Drugs Targeting Tau Protein

Tau proteins are the most abundant microtubule-associated proteins which are mainly distributed in neuronal axons and the cytoplasm [127]. In the normal brain, tau proteins help to form neurons, stabilize microtubules, and regulate anterograde transport by kinesin and neurotransmitter release. The tau hypothesis suggests that hyperphosphorylated tau proteins interfere with microtubule formation [128], causing microtubule depolymerization, neuronal synaptic dysfunction, and NFT formation. In a brain with Alzheimer’s, modifications of tau proteins’ aberrant glycosylation facilitate the subsequent hyperphosphorylation of tau [129].

Initially, studies of antitau drugs were focused on tau aggregation inhibitors and microtubule stabilizers (Table 4). However, the failure of most of them may have been due to their high toxicity or low efficacy. *TRx0237* (LMTX™) is a tau aggregation inhibitor. Its active ingredient methylthionine chloride (MTC) binds selectively to abnormal tau proteins and removes damaging tau tangles. Although it has undergone two Phase III RCTs, neither yielded positive primary results [130]. The microtubule stabilizer, *TPI-287*, causes severe hypersensitivity reactions in AD patients [131].

Another research focus in antitau therapy is the clearance of tau proteins through active or passive immunity (Table 4). One of the active immunotherapies is achieved through tau vaccine injection. The most outstanding tau vaccine, *AADvac1*, has undergone Phase II clinical trials with desirable safety and effective immune response [132]. Although improvements in cognitive functions were observed in a subgroup of patients with confirmed AD biomarker profiles, the vaccine did not slow cognitive decline in the whole study sample [133].

**Table 4:** Alzheimer drugs targeting tau protein.

Drug Name	Principle	Phase	Effect in Clinical Trials	Status	Refs.
TRx0237 (LMTM)	Inhibit Tau aggregation	Phase 3 (NCT01689233; NCT01689246; NCT02245568)	Did not significantly affect cognitive decline	Active, not recruiting	[130]
TPI-287	microtubule stabilizer	Phase 1 (NCT01966666)	Severe hypersensitivity reactions	Completed	[131]
Tilavonemab (ABBV-8E12)	Passive immunity	Phase 2 (NCT03712787; NCT02880956)	Did not change the decline of cognitive, or lower brain atrophy or levels of plasma neurofilament light	Discontinued	[134]
BIIB076 (NI-105)	Passive immunity	Phase 1 (NCT03056729)	Reduced half the amount of mid-region-bearing tau in CSF *	Discontinued	[135]
Gosuranemab (BIIB092)	Passive immunity	Phase 2 (NCT03352557)	Lack of efficacy	Discontinued	https://www.clinicaltrials.gov/ (accessed on 28 March 2023)
Semorinemab (RO07105705)	Passive immunity	Phase 2 (NCT03289143; NCT03828747)	Caused 43.6% slowed decline in the ADAS-Cog11 coprimary, and did not change tangle accumulation	Active, not recruiting	https://www.clinicaltrials.gov/ (accessed on 28 March 2023)
Bepranemab (UCB0107)	Passive immunity	Phase 2 (NCT04867616)	No drug-related adverse events or changes in safety results were reported	Active, not recruiting	[136,137]
Zagotenemab (LY3303560)	Passive immunity	Phase 2 (NCT03518073)	Missed its primary endpoint	Discontinued	[138,139]
JNJ-63733657	Passive immunity	Phase 2 (NCT04619420)	Dose-dependent reductions in free p217 tau in CSF * in volunteers. Adverse reactions: back pain and headache	Recruiting	https://www.clinicaltrials.gov/ (accessed on 28 March 2023)
AAD-vac1	Active immunity	Phase 2 (NCT02579252)	Reduced brain atrophy and cognitive decline in mild to moderate AD * patients; reduced the levels of p-tau181 and p-tau217	Completed	[132,133,140]
ACI-35	Active immunity	Phase 1 (NCT04445831)	Developed antitau IgG and IgM antibodies preferentially against phosphorylated tau, with high IgG titers	Active, not recruiting	[141]

* Abbreviation: AD, Alzheimer’s disease; CSF, cerebrospinal fluid.

Intravenous administration of antitau monoclonal antibodies reduces pathological tau protein levels through passive immunization. However, these tau antibodies fail to achieve the desired outcome. *Gosuranemab*, *Tilavonemab*, and *Zagotenemab* were discontinued after failure in Phase II RCTs, and *Semorinemab* and *Bepranemab* are still active in the development process.

### 2.3. Drugs Targeting Calcium Balance and Reactive Oxygen Species

Disrupting calcium homeostasis is a prominent feature of Alzheimer’s disease [4,142]. Several drugs targeting calcium ions (Ca^2+^) have entered clinical trials. *Memantine*, which targets the NMDAR receptor, is approved by the FDA for the treatment of moderate to severe dementia in patients with AD. *Memantine* prevents neuronal excitatory toxicity caused by excessive Ca^2+^ [143]. Drugs targeting AMPAR include *LY451395* [144], *LY450108* [145] and *S 18,986* [146], which can regulate Ca^2+^ penetration and reverse memory deficits in mice while no efficacy in cognitive improvement in AD patients are observed. The compound *Tg-2112x* protects neurons by reducing mitochondrial Ca^2+^ uptake, thereby preventing neurodegeneration and the development of dementia [147]. *Multi-target 1,4-dihydropyridines* show calcium channel blockade for AD therapy [148].

Reactive oxygen species (ROS) have been found to accelerate AD pathogenesis [149]. Several natural compounds show good antioxidant capacity. *Astaxanthin (AST)* is a potent exogenous carotenoid that can scavenge superoxide anion radicals. *AST* slows memory decline and decreases levels of Aβ and tau proteins in mice [150]. As an antioxidant neuroprotector, *AST* improves the presynaptic and postsynaptic protein markers associated with memory in APP/PS1 mice [151]. *Crocin* increases the levels of glutathione peroxidase and superoxide dismutase and reduces ROS and Aβ_1-42_ in the brain of mice [152]. *Linalool*, a monoterpene, decreases the levels of oxidative stress in AD model flies and rats [153].

### 2.4. New Hypotheses and Drug Targets for AD Treatments

Failures in AD drug developments via Aβ and tau have led to the controversy in the hypothesis of AD pathogenesis and the choice of drug targets. The most important challenge is to determine which endogenous factor directly induces Aβ aggregation and tau phosphorylation in AD. Recently, it has been proven that age-related endogenous formaldehyde is the direct trigger for Aβ- and tau-related pathology. Eliminating formaldehyde can reduce Aβ and tau aggregation and improve memory [154]. This suggests that excessive formaldehyde may be a novel target for AD therapy.

#### 2.4.1. Exogenous Formaldehyde Directly Induces AD-Like Pathology

**Figure 3:**
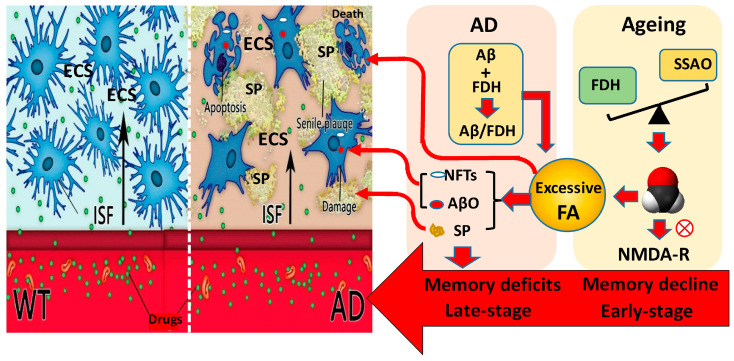
Novel pathogenesis, drug target and delivery in AD. Briefly, aging induces endogenous formaldehyde accumulation by disrupting FDH and SSAO expression [170,171]. Excessive formaldehyde can directly induce neuron death and cognitive decline by inhibiting NMDA receptor [172]. Especially, both age-associated formaldehyde and Aβ-inactivated FDH-derived formaldehyde elicit the formation of intracellular AβO and NFTs and extracellular SP [18,173,174]. Formaldehyde-induced Aβ deposition in ECS blocks ISF drainage and drug delivery [18].

Gaseous formaldehyde (FA) is widely known as an irritating toxic gas in the environment, and is particularly common in industrialized production. Certainly, occupational exposure to FA or FA solution injection can impair cognitive functions associated with hippocampal neuron death [155]. In particular, formaldehyde induces pathological manifestations similar to those in AD patients in animal brains. Formaldehyde exposure directly causes spatial memory deficits in mice accompanied by hippocampal neuron death [156] (Figure 3). Some early AD-like changes, including cognitive deficits associated with Aβ plaques and tau hyperphosphorylation, have been observed in wild-type mice after acute FA exposure [157]. Intracerebroventricular (i.c.v.) injection of formaldehyde directly induces memory impairments in young rhesus monkeys associated with SP and NFT appearance [158] (Figure 3). In addition, pathological concentrations of formaldehyde impair cognitive function by interfering with DNA methyltransferases and reducing global DNA methylation [159].

#### 2.4.2. Age-Related Endogenous Formaldehyde Induces Memory Decline

Endogenous formaldehyde is a metabolite in the human body. During aging, the imbalance in FA metabolism leads to FA accumulation and neuron death in the brain’s age-related memory decline [160]. Further, clinical investigations have demonstrated that age-related formaldehyde concentrations and memory loss are positively correlated in the elderly [161,162,163]. An increased expression of semicarbazide-sensitive amine oxidase (SSAO, a formaldehyde-generating enzyme) and the decline expression and activity of formaldehyde dehydrogenase (FDH, a formaldehyde degrading enzyme) have been proven to contribute to endogenous formaldehyde accumulation [164]. Overexpression of SSAO in the blood leads to an increase in urinary FA levels in AD patients [162]. FA and Aβ in the CSF of rhesus monkey macaques are positively correlated with age [165,166]. Brain formaldehyde levels were gradually elevated in mice during normal aging, especially, in AD model mice [167].

#### 2.4.3. Formaldehyde Elicits Aβ Oligomerization and Fibrillation

Excessive levels of the endogenous formaldehyde crosslinked nontoxic Aβ monomer promote the formation of toxic Aβ dimers, oligomers and fibrils in vitro [17]. Notably, they also increase the formation of Aβ oligomers and Aβ-related SPs associated with memory deficits in AD model mice [154]. There is direct evidence that methanol (a precursor of formaldehyde) can be oxidized to form formaldehyde in rhesus monkey brains [168]. Feeding rhesus monkeys with methanol causes an increase in the formation of SPs and sustained memory impairments [169]. Aβ also interferes with formaldehyde metabolism. For example, Aβ inactivates the FDH by binding with it, leading to formaldehyde accumulation; in turn, formaldehyde promotes Aβ oligomerization and SP formation in AD model mice [154] (Figure 3).

#### 2.4.4. Formaldehyde Promotes Tau Hyperphosphorylation and NFTs Formation

Excessive formaldehyde has been proven to elicit tau hyperphosphorylation and NFT formation via glycogen synthase kinase 3β catalysis in vitro and in vivo [173,174]. After chronic i.c.v. injections of formaldehyde, tau protein phosphorylation was observed in the hippocampus, entorhinal cortex and prefrontal cortex of rhesus macaques [158]. After feeding them with methanol for 6 months, the levels of tau protein phosphorylation on residues T181 and S396 were increased in the CSF of rhesus monkeys. Meanwhile, NFTs were also widely distributed in the brains [169].

#### 2.4.5. Endogenous Formaldehyde as a Target for AD Therapy

The above studies suggest that excessive endogenous formaldehyde directly elicits Aβ- and tau-related pathology associated with hippocampal neuron death. Thus, scavenging formaldehyde may be a potential and new method for treating AD.

*Coenzyme Q10* (CoQ10), is a vitamin-like substance that plays significant roles in the energy supply process. It has been revealed that 30 nm nanopacked Q10 (which enhances water solubility) reduces the formation of Aβ plaques and NFTs associated with improved cognitive functions in APP/PS1 mice by directly scavenging formaldehyde [154]. In addition, Q10 also reduces oxidative stress, Aβ production and intracellular Aβ deposition in the cortex of mice with a progerin 1 mutation [175].

*Resveratrol*, a natural formaldehyde scavenger, reverses formaldehyde accumulation and noradrenaline deficiency. Resveratrol can also improve LTP and memory functions in SAMP8 mice [176]. It also attenuates Tau hyperphosphorylation induced by formaldehyde in N2a cells [177]. Resveratrol reduces Aβ and p-tau pathology in the hippocampus of AD transgenic mice [178] and enhances cognitive functions in AD patients [102]. However, the low water solubility of resveratrol limits its clinical application [179], and nanopacked methods have shown a promising prospect for AD treatments [180,181].

*Epigallocatechin gallate* (EGCG), a catechin of plant origin, is primarily found in green tea. EGCG make a spontaneous reaction with formaldehyde at room temperature (25 °C) in vitro [182]. A large number of studies support the viewpoint that EGCG has potential neuroprotective effects in neurological diseases including AD [183]. EGCG also activates the Nrf2 signaling pathway and reduces formaldehyde-induced oxidative stress [184]. EGCG reduces Aβ deposition and phosphorylated tau and improves learning and memory in AD mice [185,186,187]. Although the low water solubility of resveratrol limits its clinical application, the nanopacked method has shown a promise prospect for AD treatments.

*Hydrogen Sulfide* (H_2_S), a signaling molecule, is associated with several systemic diseases, including AD [188,189]. A study found that sodium hydrosulfide, a donor of H_2_S, markedly scavenges formaldehyde, increases hippocampal brain-derived neurotrophic factor expression, and alleviates cognitive deficits in formaldehyde-exposed rats [190]. H_2_S reduces Aβ_1-42_ production by inhibiting APP expression promoted by exogenous ATP [191], and improves cognitive function in AD models [192,193,194]. Whether this gaseous molecule has clinical effects for AD is unclear.

#### 2.4.6. Formaldehyde-Degrading Enzyme-ALDH2 as a Target for AD Treatments

Aldehyde dehydrogenase 2 (ALDH2, a formaldehyde-degrading enzyme) is expressed at the highest levels in the liver, kidneys, muscles and the heart, while it is less expressed in the brain [195]. ALDH2 genetic polymorphism is associated with many diseases including aortic aneurysm/dissection (AAD), hypertension, liver disease and cancer [196,197,198,199]. In alcohol-related diseases, ALDH2-deficient individuals are more susceptible to endogenous formaldehyde [200]. The most common genetic mutation is ALDH2*2 associated with cognitive impairment [201,202]. A meta-analysis has shown that the polymorphic locus rs671 G > A of ALDH2 is a potential risk factor for AD in East Asians. An allele mutation results in inactivated ALDH2 proteins, which may explain why carriers of the AA allele are more likely to develop AD than carriers of the GG allele are [203,204]. Toxic aldehydes accumulated in ALDH2-deficient mice brains induce Aβ plaques and NFT formation associated with cognitive impairments [205,206]. On the contrary, ALDH2 overexpression not only reverses cognitive deficits, but also improves mitochondrial integrity and neuronal survival by reducing aldehyde and Aβ toxicity [207].

*Alda-1,* an ALDH2 activator, significantly protects neurovascular cells from excessive formaldehyde during AD progression [167]. Alda-1 also protects against Aβ toxicity, neuroinflammation [208], and Aβ-induced mitochondrial geometry anomalies [207].

#### 2.4.7. Formaldehyde-Degrading Enzyme-ALDH2 as a Target for AD Treatments

A previous study revealed that red light at 630 nm can penetrate the skulls of mice, and not only reduces levels of H_2_O_2_ in the brain, but also activates FDH (a specific formaldehyde-degrading enzyme [164]) and scavenges excessive formaldehyde in the brains. Subsequently, FDH activation by red light can alleviate memory deficits in AD model mice [18,171].

## 3. Enhancing BBB Penetration for Drug Delivery in AD

Although the BBB is a physical barrier to drug delivery, carrier-mediated transport is the approach through the BBB for small molecules, carbohydrates, amino acids, fatty acids, and ions. Receptor-mediated transcytosis is finding a principal pathway for macromolecules, including proteins and peptides, to enter the central nervous system [209]. In Alzheimer’s brains, the structure and function of the BBB are disrupted [210]. Aβ induces astrocyte endfeet retraction leading to neurovascular uncoupling [211,212]. A reduction in pericytes has been observed in the cortex and hippocampus [213], leading to a lower clearance of soluble Aβ in interstitial fluid and accelerated brain pathology changes [214]. Therapeutic drugs tend to be trapped in the enlarged perivascular space, which makes it difficult for them to be diffused through brain ECS to reach injured neurons [8] (Figure 3).

The application of nanopacked medicines facilitates the entry of drugs through the BBB. Several nanomedicines have been developed based on alterations in the BBB during disease. Because the expression of the receptor of advanced glycation endproducts (RAGE) in the microvasculature increases [215], RAGE-mediated transcytosis can be used to deliver drugs to brains with Alzheimer’s. For example, an ibuprofen and FK506-encapsulated drug codelivery system (Ibu&FK@RNPs) targeting RAGE inhibits the neuroinflammation caused by the NF-κB pathway [216].

## 4. Aβ Plaques Deposition in ECS Blocks Drug Delivery in AD

Interstitial fluid (ISF) drainage is necessary for drug delivery to target neurons in the brain. The myelin sheath separates the normal brain into different regions, affecting ISF drainage and causing an uneven distribution of drugs in the brain. For example, ISF in the caudate nucleus flows smoothly without being blocked along myelinated fiber tracts toward the ipsilateral cortex, while ISF flowing in the opposite direction is completely blocked by barrier structures [16].

In 2012, a method to visually detect brain ISF drainage was established. The dynamic process of ISF drainage in rat brains can be imaged by magnetic resonance imaging (MRI) with gadolinium-diethylenetriaminepentaacetic acid (Gd-DTPA) as the tracer [217]. The diffusion properties of extracellular space (ECS) are usually evaluated in terms of volume fraction (α) and tortuosity (λ), with α being the ratio of the volume of ECS to the total volume of brain tissue, and λ being the ratio of the actual distance between two points to the distance between straight lines. α and λ describe the geometric characteristics of the cases in which the ECS can be used for diffusion; i.e., they describe the magnitude factors that impede the diffusion of molecules. In normal brain tissue, the ECS has a volume fraction of about 20% with a tortuosity of about 1.6 [218]. ISF drainage flows from the superficial cortex and then deep into brain to the 3rd ventricle (V3) pouring into the cerebrospinal fluid (CSF). Finally, CSF flows into nasal lymphatics (NL) where the substances are exchanged with blood [18] (Figure 2). The diffusion function of the ECS is disturbed in pathological conditions. One of the typical AD pathological features is Aβ deposition in brain ECS [219]. Aβ plaques in the ECS impede the drainage of ISF from the superficial to the deeper cortical layers and drive the diffusion of ISF around neurons (in a horizontal direction) [18] (Figure 3). Aβ plaque and glial cell proliferation in AD mice leads to ECS volume elevation and ISF flow restriction [220,221], which make it difficult for drugs to reach the deeper layers of the brain. Meanwhile, toxic metabolite accumulation exacerbates deep neuronal apoptosis or death. This may be a possible explanation for the AD drug development failures over the last hundred years.

## 5. Novel Drug Delivery for AD Treatments

### 5.1. Drug Delivery via Brain ECS

**Figure 4:**
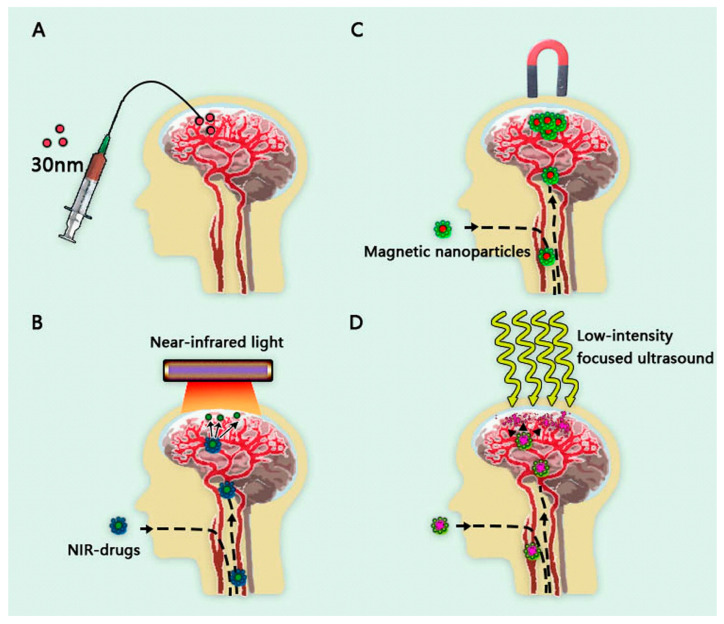
Direction of drug development for Alzheimer’s disease. (**A**). Drug delivery via brain ECS. (**B**). Magnetic nanoparticles [223]. (**C**). Near-infrared photosensitive nanomedicines [230]. (**D**). Combination of focused ultrasound and nanomedicines [238].

Drug delivery via brain ECS involves direct therapeutic drug injection into damaged deep neurons through a specialized catheter, avoiding a route with slow or less ISF drainage which results in reduced drug concentrations and low clinical efficacy (Figure 4A). MRI-guided stereotactic delivery improves the neuroprotective efficiency of drugs [222]. Although it is a promising method for AD drug delivery, this invasive treatment poses the risk of intracranial infection and hemorrhage to patients. Additionally, it requires detailed study of the locations and functions of brain subdivision before a highly precise procedure can be performed.

### 5.2. Magnetic Nanoparticles

Magnetic nanoparticles (MNPs) can effectively penetrate the BBB and reach specific brain regions when exposed to external magnetic fields [223,224] (Figure 4B). Superparamagnetic iron oxide nanoparticles (SPION) are currently the focus of research, and have the advantages of inherent magnetism, high safety and targeting, and easy access to manufacturing methods [225,226]. For example, Tween 80-modified SPION (Tween-SPIONs), a kind of MNP, can pass through the BBB in rats and accumulates in large amounts in the cortex near the magnet [227]. In the presence of an external static magnetic field (SMF), insulin-modified NPs can effectively cross the BBB and improve the bioavailability of insulin in the brain [228].

### 5.3. Near-Infrared Photosensitive Nanomedicines

Near-infrared light (NIR) is an electromagnetic wave between visible light and mid-infrared light. The permeability and low tissue destruction of NIR make it widely used in biological science and related fields, particularly in brain imaging [229] and nanomedicine therapy [230] (Figure 4C). NIR combined with nanomedicine is commonly used in antitumor therapy. For example, a NIR-excitable immunomodulating nanophotosensitizer has been developed as an effective and precise antitumor immunotherapy [231]. NIR-based phototherapy, a nanoplatform of the brain-targeting peptide RVG conjugated with the 2D porphyrinic PCN-222 metal–organic framework and indocyanine green (PCN-222@ICG@RVG), has been established for inhibiting Aβ aggregation by NIR irradiation [232]. Local photothermal heat facilitates the photo-oxygenation process of generating oxidized Aβ monomers with low aggregation capability [232]. Human serum albumin-stabilized gold nanoclusters (AuNCs@HSA) have been found to inhibit Aβ aggregation, oxidize Aβ monomers, and attenuate Aβ-mediated neurotoxicity through photo-oxidation under NIR laser irradiation [233].

### 5.4. Combination of Focused Ultrasound and Nanomedicines

Focused ultrasound therapy is characterized by high tissue penetration and submillimeter steerable focusing. MRI-guided low-intensity focused ultrasound (FUS) serves to induce the BBB to open safely, noninvasively, transiently and centrally in the human hippocampus and internal olfactory cortex [234]. Some studies have revealed that increases in brain interstitial fluid and lymphatic drainage and the opening of the BBB by FUS reduce Aβ plaques [235,236]. FUS applied with microbubbles (FUS^+MB^) is a novel technique used to breach the BBB and increase drug delivery. After FUS^+MB^ treatment, the delivery of two therapeutic AD antibodies, Aducanumab and RNF5, increases significantly [237].

FUS allows nanomedicines to be released in a specific brain region (Figure 4D). For example, an albumin-based nanocluster and the FUS facilitate the opening of the BBB, allowing the nanocluster to enter the ECS. After localization of drug using MRI, a second FUS will release the nanocluster into the brain tissue [238]. Another method of the embedded combining quercetin modified sulfur nanoparticles (Qc@SNPs) into microbubbles (MB) construct a Qc@SNPs-MB nanosystem. FUS helps to release drugs embedded in microbubbles and cross the transiently opened the BBB, thus improve the abilities of learning and memory in AD mice [239].

### 5.5. Extracellular Vesicles

Extracellular vesicles (EVs) are particles that are naturally released from cells [240]. It was found that tumor-derived EVs can breach an intact BBB during brain metastasis [241]. Rabies viral glycoprotein-tagged exosomes derived from Mesenchymal stem cells (RVG-tagged MSC-Exo) decrease plaque deposition and Aβ and prevent memory deficits in APP/PS1 mice [242].

### 5.6. BBB Shuttle Peptide

The BBB shuttle peptide is used to increase the ability of adeno-associated virus (AAV) vectors to cross the BBB [243]. A study showed that the PB5-3 peptide increased AAV9 transport and transendocytosis efficiency [244].

## 6. Conclusions and Outlook

For the past two decades, many Alzheimer’s disease drug candidates have failed in trials. Although these drugs showed some successes in cellular and animal model experiments, they did not improve cognitive functions in clinical Alzheimer’s patients. Aβ monoclonal antibodies reduce Aβ levels in brains with adverse reactions, especially ARIA. Aβ deposition in blood vessels, brain extracellular space, an impaired BBB and blocked ISF drainage cause low efficacy drug delivery. This leads to the lack of therapeutic efficacy in AD drugs. Therefore, there is an imperative need for new therapies that increase BBB permeability and ISF drainage. Encouragingly, red light, near-infrared and focused ultrasound have been proven to enhance ISF drainage in brain ECS [18,235,245].

Even though drug delivery via brain ECS increases precision, it carries infection and intracerebral hemorrhage risks. The noninvasive, low toxicity and high targeting characteristics of physical therapy, nanomedicines with NIR and/or focused ultrasound are considered to be the promising methods. In addition, endogenous formaldehyde is proposed to be a direct endogenous factor in intracellular Aβ oligomerization, NFT formation, and Aβ deposition in ECS in AD. Red light therapy at 630 nm can activate FDH to degrade formaldehyde, smash Aβ plaques, increase ISF flow to deep into the brain, and improve cognitive functions in AD models [18]. Thus, nanopackaged medicines targeting formaldehyde for reducing SP, and new physical methods for accelerating ISF drainage may be the promising strategies for clinical AD therapy.

## Figures and Tables

**Table 2:** Alzheimer drugs used to prevent Aβ aggregation.

Drug Name	Principle	Phase	Effect in Clinical Trials	Status	Refs.
PBT2	Reduction in Aβ aggregation	Phase 2 (NCT01590888)	The higher dose reportedly reduced Aβ_42_ levels in CSF *	Completed	[99,100]
Resveratrol	Anti-oxidant capacity; prevention of amyloid deposition	Phase 3 (NCT01504854)	Reduce cognitive impairment and Aβ_42_ in CSF *; increased Aβ_40_ levels in CSF * and plasma; increased brain volume loss	Withdraw	[101,102]
Alzhemed™ (Tramiprosate)	Inhibit the interaction of Aβ with endogenous glycosaminoglycans	Phase 3 (NCT00314912)	Slowed cognitive decline in ApoE4 homozygotes	Unknown	[89,103]
Epigallocatechin Gallate	Remodel toxic amyloid-beta fibrils	Phase 2/3 (NCT00951834)	No public information	Completed	[47]

* Abbreviations: CSF, cerebrospinal fluid.

**Table 3:** Alzheimer drugs to promote Aβ clearance.

Drug Name	Principle	Phase	Effect in Clinical Trials	Status	Refs.
Aducanumab (BIIB037)	Passive immunity	Phase 3 (NCT02484547; NCT02477800; NCT01677572)	Bound to soluble monomeric Aβ and reduce brain Aβ; reduced cognitive impairment only at the highest dose; adverse reactions: ARIA *	Approved	[106,107,108]
Lecanemab (BAN2401)	Passive immunity	Phase 3 (NCT04468659; NCT03887455)	Reduced markers of amyloid in early AD * Alleviated cognitive and functional decline; adverse reactions: ARIA *, infusion-related reactions	Approved	[20,109,110]
Remternetug (LY3372993)	Passive immunity	Phase 3 (NCT05463731)	No public information	Recruiting	https://www.clinicaltrials.gov/ (accessed on 28 March 2023)
Gantenerumab (RO4909832)	Passive immunity	Phase 3 (NCT04339413; NCT04339413; NCT02051608)	No reduction in cognitive impairment; adverse reactions: ARIA *	Terminated	[111,112]
Solanezumab (LY2062430)	Passive immunity	Phase 3 (NCT02760602; NCT01900665; NCT01127633)	Did not significantly affect cognitive decline	Terminated	[112,113]
Crenezumab (MABT5102A)	Passive immunity	Phase 3 (NCT03491150; NCT03114657; NCT03114657)	Did not reduce cognitive decline in participants with early AD *	Terminated	[114,115]
Donanemab (LY30028123)	Passive immunity	Phase 2 (NCT03367403)	Improved cognition and daily living ability in early AD patients; reduce amyloid plaque levels and overall tau load	Recruiting	[116,117,118,119,120]
ABvac40	Active immunity	Phase 1 (NCT03113812)	Good safety and tolerance; triggered a consistent and specific immune response	Unknown	[121]
ACI-24	Active immunity	Phase 2 (2018-000445-39)	Produced a low IgG antibody response, increased CSF * Aβ_40_ and Aβ_42_ levels but caused no change in amyloid-PET.	Completed	https://www.clinicaltrialsregister.eu/ (accessed on 28 March 2023)
Amilomotide (CAD106)	Active immunity	Phase 2 Phase 3 (NCT00795418)	Unexpected changes in cognitive function, brain volume loss, and body weight loss	Terminated	[122]
UB-311	Active immunity	Phase 2 (NCT03531710; NCT03531710)	A slower rate of increase in ADAS-Cog in mild AD * patients; 100% responder rate	Completed	[123]

* Abbreviations: AD, Alzheimer’s disease; CSF, cerebrospinal fluid; ARIA, amyloid-related imaging abnormalities.

## Data Availability

Not applicable.

## References

[1] Alzheimer’s Association (2016). 2016 Alzheimer’s disease facts and figures. Alzheimers Dement..

[2] Gu Z., Chen H., Zhao H., Yang W., Song Y., Li X., Wang Y., Du D., Liao H., Pan W. (2022). New insight into brain disease therapy: Nanomedicines-crossing blood-brain barrier and extracellular space for drug delivery. Expert. Opin. Drug. Deliv..

[3] Tiwari S., Atluri V., Kaushik A., Yndart A., Nair M. (2019). Alzheimer’s disease: Pathogenesis, diagnostics, and therapeutics. Int. J. Nanomed..

[4] Tong B.C., Wu A.J., Li M., Cheung K.H. (2018). Calcium signaling in Alzheimer’s disease & therapies. Biochim. Biophys. Acta Mol. Cell. Res..

[5] Bai R., Guo J., Ye X.Y., Xie Y., Xie T. (2022). Oxidative stress: The core pathogenesis and mechanism of Alzheimer’s disease. Ageing Res. Rev..

[6] Liebner S., Dijkhuizen R.M., Reiss Y., Plate K.H., Agalliu D., Constantin G. (2018). Functional morphology of the blood-brain barrier in health and disease. Acta Neuropathol..

[7] Banks W.A. (2016). From blood-brain barrier to blood-brain interface: New opportunities for CNS drug delivery. Nat. Rev. Drug. Discov..

[8] Sweeney M.D., Sagare A.P., Zlokovic B.V. (2018). Blood-brain barrier breakdown in Alzheimer disease and other neurodegenerative disorders. Nat. Rev. Neurol..

[9] Kong C., Yang E.J., Shin J., Park J., Kim S.H., Park S.W., Chang W.S., Lee C.H., Kim H., Kim H.S. (2022). Enhanced delivery of a low dose of aducanumab via FUS in 5×FAD mice, an AD model. Transl. Neurodegener..

[10] Shamsi A., Shahwan M., Alhumaydhi F.A., Alwashmi A.S.S., Aljasir M.A., Alsagaby S.A., Al Abdulmonem W., Hassan M.I., Islam A. (2021). Spectroscopic, calorimetric and in silico insight into the molecular interactions of Memantine with human transferrin: Implications of Alzheimer’s drugs. Int. J. Biol. Macromol..

[11] Khan M.S., Shahwan M., Shamsi A., Alhumaydhi F.A., Alsagaby S.A., Al Abdulmonem W., Abdullaev B., Yadav D.K. (2022). Elucidating the Interactions of Fluoxetine with Human Transferrin Employing Spectroscopic, Calorimetric, and In Silico Approaches: Implications of a Potent Alzheimer’s Drug. ACS Omega.

[12] Andrade S., Loureiro J.A., Pereira M.C. (2022). Transferrin-Functionalized Liposomes for the Delivery of Gallic Acid: A Therapeutic Approach for Alzheimer’s Disease. Pharmaceutics.

[13] Nicholson C., Hrabětová S. (2017). Brain Extracellular Space: The Final Frontier of Neuroscience. Biophys. J..

[14] Hrabetova S., Cognet L., Rusakov D.A., Nägerl U.V. (2018). Unveiling the Extracellular Space of the Brain: From Super-resolved Microstructure to In Vivo Function. J. Neurosci..

[15] Plog B.A., Nedergaard M. (2018). The Glymphatic System in Central Nervous System Health and Disease: Past, Present, and Future. Annu. Rev. Pathol..

[16] Wang A., Wang R., Cui D., Huang X., Yuan L., Liu H., Fu Y., Liang L., Wang W., He Q. (2019). The Drainage of Interstitial Fluid in the Deep Brain is Controlled by the Integrity of Myelination. Aging Dis..

[17] Chen K., Maley J., Yu P.H. (2006). Potential inplications of endogenous aldehydes in beta-amyloid misfolding, oligomerization and fibrillogenesis. J. Neurochem..

[18] Yue X., Mei Y., Zhang Y., Tong Z., Cui D., Yang J., Wang A., Wang R., Fei X., Ai L. (2019). New insight into Alzheimer’s disease: Light reverses Aβ-obstructed interstitial fluid flow and ameliorates memory decline in APP/PS1 mice. Alzheimer’s Dement. Transl. Res. Clin. Interv..

[19] Hong S., Quintero-Monzon O., Ostaszewski B.L., Podlisny D.R., Cavanaugh W.T., Yang T., Holtzman D.M., Cirrito J.R., Selkoe D.J. (2011). Dynamic analysis of amyloid β-protein in behaving mice reveals opposing changes in ISF versus parenchymal Aβ during age-related plaque formation. J. Neurosci..

[20] Van Dyck C.H., Swanson C.J., Aisen P., Bateman R.J., Chen C., Gee M., Kanekiyo M., Li D., Reyderman L., Cohen S. (2023). Lecanemab in Early Alzheimer’s Disease. N. Engl. J. Med..

[21] Conti Filho C.E., Loss L.B., Marcolongo-Pereira C., Rossoni Junior J.V., Barcelos R.M., Chiarelli-Neto O., da Silva B.S., Passamani Ambrosio R., Castro F., Teixeira S.F. (2023). Advances in Alzheimer’s disease’s pharmacological treatment. Front. Pharmacol..

[22] Dhillon S. (2021). Aducanumab: First Approval. Drugs.

[23] Hoy S.M. (2023). Lecanemab: First Approval. Drugs.

[24] Imbimbo B.P., Ippati S., Watling M., Balducci C. (2021). Accelerating Alzheimer’s disease drug discovery and development: What’s the way forward?. Expert. Opin. Drug. Discov..

[25] Haass C., Kaether C., Thinakaran G., Sisodia S. (2012). Trafficking and proteolytic processing of APP. Cold Spring Harb. Perspect. Med..

[26] Jeong H., Shin H., Hong S., Kim Y. (2022). Physiological Roles of Monomeric Amyloid-β and Implications for Alzheimer’s Disease Therapeutics. Exp. Neurobiol..

[27] Zheng H., Koo E.H. (2006). The amyloid precursor protein: Beyond amyloid. Mol. Neurodegener..

[28] Hardy J., Selkoe D.J. (2002). The amyloid hypothesis of Alzheimer’s disease: Progress and problems on the road to therapeutics. Science.

[29] Gu L., Guo Z. (2013). Alzheimer’s Aβ42 and Aβ40 peptides form interlaced amyloid fibrils. J. Neurochem..

[30] Yamin G. (2009). NMDA receptor-dependent signaling pathways that underlie amyloid beta-protein disruption of LTP in the hippocampus. J. Neurosci. Res..

[31] Beckman D., Ott S., Donis-Cox K., Janssen W.G., Bliss-Moreau E., Rudebeck P.H., Baxter M.G., Morrison J.H. (2019). Oligomeric Aβ in the monkey brain impacts synaptic integrity and induces accelerated cortical aging. Proc. Natl. Acad. Sci. USA.

[32] Thal D.R., Walter J., Saido T.C., Fändrich M. (2015). Neuropathology and biochemistry of Aβ and its aggregates in Alzheimer’s disease. Acta Neuropathol..

[33] Yuan Q., Xian Y.F., Huang Y.F., Wu W., Song Y.Q., Lin Z.X. (2020). Intracisternal injection of beta-amyloid seeds promotes cerebral amyloid angiopathy. Brain Behav. Immun..

[34] Zhang Y., Zhang Y., Aman Y., Ng C.T., Chau W.H., Zhang Z., Yue M., Bohm C., Jia Y., Li S. (2021). Amyloid-β toxicity modulates tau phosphorylation through the PAX6 signalling pathway. Brain.

[35] Kennedy M.E., Stamford A.W., Chen X., Cox K., Cumming J.N., Dockendorf M.F., Egan M., Ereshefsky L., Hodgson R.A., Hyde L.A. (2016). The BACE1 inhibitor verubecestat (MK-8931) reduces CNS β-amyloid in animal models and in Alzheimer’s disease patients. Sci. Transl. Med..

[36] Egan M.F., Kost J., Tariot P.N., Aisen P.S., Cummings J.L., Vellas B., Sur C., Mukai Y., Voss T., Furtek C. (2018). Randomized Trial of Verubecestat for Mild-to-Moderate Alzheimer’s Disease. N. Engl. J. Med..

[37] Egan M.F., Kost J., Voss T., Mukai Y., Aisen P.S., Cummings J.L., Tariot P.N., Vellas B., van Dyck C.H., Boada M. (2019). Randomized Trial of Verubecestat for Prodromal Alzheimer’s Disease. N. Engl. J. Med..

[38] Sur C., Kost J., Scott D., Adamczuk K., Fox N.C., Cummings J.L., Tariot P.N., Aisen P.S., Vellas B., Voss T. (2020). BACE inhibition causes rapid, regional, and non-progressive volume reduction in Alzheimer’s disease brain. Brain.

[39] Wang H., Li R., Shen Y. (2013). β-Secretase: Its biology as a therapeutic target in diseases. Trends Pharmacol. Sci..

[40] Hur J.Y. (2022). γ-Secretase in Alzheimer’s disease. Exp. Mol. Med..

[41] Doody R.S., Raman R., Farlow M., Iwatsubo T., Vellas B., Joffe S., Kieburtz K., He F., Sun X., Thomas R.G. (2013). A phase 3 trial of semagacestat for treatment of Alzheimer’s disease. N. Engl. J. Med..

[42] Coric V., Salloway S., van Dyck C.H., Dubois B., Andreasen N., Brody M., Curtis C., Soininen H., Thein S., Shiovitz T. (2015). Targeting Prodromal Alzheimer Disease with Avagacestat: A Randomized Clinical Trial. JAMA Neurol..

[43] Penninkilampi R., Brothers H.M., Eslick G.D. (2016). Pharmacological Agents Targeting γ-Secretase Increase Risk of Cancer and Cognitive Decline in Alzheimer’s Disease Patients: A Systematic Review and Meta-Analysis. J. Alzheimers Dis..

[44] Wilcock G.K., Black S.E., Hendrix S.B., Zavitz K.H., Swabb E.A., Laughlin M.A. (2008). Efficacy and safety of tarenflurbil in mild to moderate Alzheimer’s disease: A randomised phase II trial. Lancet Neurol..

[45] Green R.C., Schneider L.S., Amato D.A., Beelen A.P., Wilcock G., Swabb E.A., Zavitz K.H. (2009). Effect of tarenflurbil on cognitive decline and activities of daily living in patients with mild Alzheimer disease: A randomized controlled trial. JAMA.

[46] Vingtdeux V., Marambaud P. (2012). Identification and biology of α-secretase. J. Neurochem..

[47] Manzine P.R., Ettcheto M., Cano A., Busquets O., Marcello E., Pelucchi S., Di Luca M., Endres K., Olloquequi J., Camins A. (2019). ADAM10 in Alzheimer’s disease: Pharmacological modulation by natural compounds and its role as a peripheral marker. Biomed. Pharmacother..

[48] Reinhardt S., Stoye N., Luderer M., Kiefer F., Schmitt U., Lieb K., Endres K. (2018). Identification of disulfiram as a secretase-modulating compound with beneficial effects on Alzheimer’s disease hallmarks. Sci. Rep..

[49] Etcheberrigaray R., Tan M., Dewachter I., Kuipéri C., Van der Auwera I., Wera S., Qiao L., Bank B., Nelson T.J., Kozikowski A.P. (2004). Therapeutic effects of PKC activators in Alzheimer’s disease transgenic mice. Proc. Natl. Acad. Sci. USA.

[50] Nelson T.J., Sun M.K., Lim C., Sen A., Khan T., Chirila F.V., Alkon D.L. (2017). Bryostatin Effects on Cognitive Function and PKCɛ in Alzheimer’s Disease Phase IIa and Expanded Access Trials. J. Alzheimers Dis..

[51] Endres K., Fahrenholz F., Lotz J., Hiemke C., Teipel S., Lieb K., Tüscher O., Fellgiebel A. (2014). Increased CSF APPs-α levels in patients with Alzheimer disease treated with acitretin. Neurology.

[52] Ettcheto M., Cano A., Manzine P.R., Busquets O., Verdaguer E., Castro-Torres R.D., García M.L., Beas-Zarate C., Olloquequi J., Auladell C. (2020). Epigallocatechin-3-Gallate (EGCG) Improves Cognitive Deficits Aggravated by an Obesogenic Diet Through Modulation of Unfolded Protein Response in APPswe/PS1dE9 Mice. Mol. Neurobiol..

[53] Obregon D.F., Rezai-Zadeh K., Bai Y., Sun N., Hou H., Ehrhart J., Zeng J., Mori T., Arendash G.W., Shytle D. (2006). ADAM10 activation is required for green tea (-)-epigallocatechin-3-gallate-induced alpha-secretase cleavage of amyloid precursor protein. J. Biol. Chem..

[54] Bao J., Liu W., Zhou H.Y., Gui Y.R., Yang Y.H., Wu M.J., Xiao Y.F., Shang J.T., Long G.F., Shu X.J. (2020). Epigallocatechin-3-gallate Alleviates Cognitive Deficits in APP/PS1 Mice. Curr. Med. Sci..

[55] Pervin M., Unno K., Ohishi T., Tanabe H., Miyoshi N., Nakamura Y. (2018). Beneficial Effects of Green Tea Catechins on Neurodegenerative Diseases. Molecules.

[56] Mei Z., Situ B., Tan X., Zheng S., Zhang F., Yan P., Liu P. (2010). Cryptotanshinione upregulates alpha-secretase by activation PI3K pathway in cortical neurons. Brain Res..

[57] Durairajan S.S., Liu L.F., Lu J.H., Koo I., Maruyama K., Chung S.K., Huang J.D., Li M. (2011). Stimulation of non-amyloidogenic processing of amyloid-β protein precursor by cryptotanshinone involves activation and translocation of ADAM10 and PKC-α. J. Alzheimers Dis..

[58] Kuang X., Zhou H.J., Thorne A.H., Chen X.N., Li L.J., Du J.R. (2017). Neuroprotective Effect of Ligustilide through Induction of α-Secretase Processing of Both APP and Klotho in a Mouse Model of Alzheimer’s Disease. Front. Aging Neurosci..

[59] Shi C., Zheng D.D., Wu F.M., Liu J., Xu J. (2012). The phosphatidyl inositol 3 kinase-glycogen synthase kinase 3β pathway mediates bilobalide-induced reduction in amyloid β-peptide. Neurochem. Res..

[60] Yin Y., Ren Y., Wu W., Wang Y., Cao M., Zhu Z., Wang M., Li W. (2013). Protective effects of bilobalide on Aβ(25–35) induced learning and memory impairments in male rats. Pharmacol. Biochem. Behav..

[61] Narasingappa R.B., Javagal M.R., Pullabhatla S., Htoo H.H., Rao J.K., Hernandez J.F., Govitrapong P., Vincent B. (2012). Activation of α-secretase by curcumin-aminoacid conjugates. Biochem. Biophys. Res. Commun..

[62] Reddy P.H., Manczak M., Yin X., Grady M.C., Mitchell A., Tonk S., Kuruva C.S., Bhatti J.S., Kandimalla R., Vijayan M. (2018). Protective Effects of Indian Spice Curcumin Against Amyloid-β in Alzheimer’s Disease. J. Alzheimers Dis..

[63] Lopez Lopez C., Tariot P.N., Caputo A., Langbaum J.B., Liu F., Riviere M.E., Langlois C., Rouzade-Dominguez M.L., Zalesak M., Hendrix S. (2019). The Alzheimer’s Prevention Initiative Generation Program: Study design of two randomized controlled trials for individuals at risk for clinical onset of Alzheimer’s disease. Alzheimer’s Dement. Transl. Res. Clin. Interv..

[64] Neumann U., Ufer M., Jacobson L.H., Rouzade-Dominguez M.L., Huledal G., Kolly C., Lüönd R.M., Machauer R., Veenstra S.J., Hurth K. (2018). The BACE-1 inhibitor CNP520 for prevention trials in Alzheimer’s disease. EMBO Mol. Med..

[65] Luo X., Yan R. (2010). Inhibition of BACE1 for therapeutic use in Alzheimer’s disease. Int. J. Clin. Exp. Pathol..

[66] Ghosh A.K., Brindisi M., Tang J. (2012). Developing β-secretase inhibitors for treatment of Alzheimer’s disease. J. Neurochem..

[67] May P.C., Dean R.A., Lowe S.L., Martenyi F., Sheehan S.M., Boggs L.N., Monk S.A., Mathes B.M., Mergott D.J., Watson B.M. (2011). Robust central reduction of amyloid-β in humans with an orally available, non-peptidic β-secretase inhibitor. J. Neurosci..

[68] May P.C., Willis B.A., Lowe S.L., Dean R.A., Monk S.A., Cocke P.J., Audia J.E., Boggs L.N., Borders A.R., Brier R.A. (2015). The potent BACE1 inhibitor LY2886721 elicits robust central Aβ pharmacodynamic responses in mice, dogs, and humans. J. Neurosci..

[69] Quartino A., Huledal G., Sparve E., Lüttgen M., Bueters T., Karlsson P., Olsson T., Paraskos J., Maltby J., Claeson-Bohnstedt K. (2014). Population pharmacokinetic and pharmacodynamic analysis of plasma Aβ40 and Aβ42 following single oral doses of the BACE1 inhibitor AZD3839 to healthy volunteers. Clin. Pharmacol. Drug. Dev..

[70] Forman M., Tseng J., Palcza J., Leempoels J., Ramael S., Krishna G., Ma L., Wagner J., Troyer M. (2012). The Novel BACE Inhibitor MK-8931 Dramatically Lowers CSF Aβ Peptides in Healthy Subjects: Results from a Rising Single Dose Study (PL02.004). Neurology.

[71] Wessels A.M., Tariot P.N., Zimmer J.A., Selzler K.J., Bragg S.M., Andersen S.W., Landry J., Krull J.H., Downing A.M., Willis B.A. (2020). Efficacy and Safety of Lanabecestat for Treatment of Early and Mild Alzheimer Disease: The AMARANTH and DAYBREAK-ALZ Randomized Clinical Trials. JAMA Neurol..

[72] Lai R., Albala B., Kaplow J.M., Aluri J., Yen M., Satlin A. (2012). O1-06-05: First-in-human study of E2609, a novel BACE1 inhibitor, demonstrates prolonged reductions in plasma beta-amyloid levels after single dosing. Alzheimer’s Dement..

[73] Albala B., Kaplow J.M., Lai R., Matijevic M., Aluri J., Satlin A. (2012). S4-04-01: CSF amyloid lowering in human volunteers after 14 days’ oral administration of the novel BACE1 inhibitor E2609. Alzheimer’s Dement..

[74] Timmers M., Van Broeck B., Ramael S., Slemmon J., De Waepenaert K., Russu A., Bogert J., Stieltjes H., Shaw L.M., Engelborghs S. (2016). Profiling the dynamics of CSF and plasma Aβ reduction after treatment with JNJ-54861911, a potent oral BACE inhibitor. Alzheimer’s Dement. Transl. Res. Clin. Interv..

[75] Timmers M., Streffer J.R., Russu A., Tominaga Y., Shimizu H., Shiraishi A., Tatikola K., Smekens P., Börjesson-Hanson A., Andreasen N. (2018). Pharmacodynamics of atabecestat (JNJ-54861911), an oral BACE1 inhibitor in patients with early Alzheimer’s disease: Randomized, double-blind, placebo-controlled study. Alzheimers Res. Ther..

[76] Sperling R., Henley D., Aisen P.S., Raman R., Donohue M.C., Ernstrom K., Rafii M.S., Streffer J., Shi Y., Karcher K. (2021). Findings of Efficacy, Safety, and Biomarker Outcomes of Atabecestat in Preclinical Alzheimer Disease: A Truncated Randomized Phase 2b/3 Clinical Trial. JAMA Neurol..

[77] Novak G., Streffer J.R., Timmers M., Henley D., Brashear H.R., Bogert J., Russu A., Janssens L., Tesseur I., Tritsmans L. (2020). Long-term safety and tolerability of atabecestat (JNJ-54861911), an oral BACE1 inhibitor, in early Alzheimer’s disease spectrum patients: A randomized, double-blind, placebo-controlled study and a two-period extension study. Alzheimers Res. Ther..

[78] Koriyama Y., Hori A., Ito H., Yonezawa S., Baba Y., Tanimoto N., Ueno T., Yamamoto S., Yamamoto T., Asada N. (2021). Discovery of Atabecestat (JNJ-54861911): A Thiazine-Based β-Amyloid Precursor Protein Cleaving Enzyme 1 Inhibitor Advanced to the Phase 2b/3 EARLY Clinical Trial. J. Med. Chem..

[79] Willis B.A., Lowe S.L., Monk S.A., Cocke P.J., Aluise C.D., Boggs L.N., Borders A.R., Brier R.A., Dean R.A., Green S.J. (2022). Robust Pharmacodynamic Effect of LY3202626, a Central Nervous System Penetrant, Low Dose BACE1 Inhibitor, in Humans and Nonclinical Species. J. Alzheimers Dis. Rep..

[80] Lo A.C., Evans C.D., Mancini M., Wang H., Shcherbinin S., Lu M., Natanegara F., Willis B.A. (2021). Phase II (NAVIGATE-AD study) Results of LY3202626 Effects on Patients with Mild Alzheimer’s Disease Dementia. J. Alzheimers Dis. Rep..

[81] Mitani Y., Yarimizu J., Saita K., Uchino H., Akashiba H., Shitaka Y., Ni K., Matsuoka N. (2012). Differential effects between γ-secretase inhibitors and modulators on cognitive function in amyloid precursor protein-transgenic and nontransgenic mice. J. Neurosci..

[82] Coric V., van Dyck C.H., Salloway S., Andreasen N., Brody M., Richter R.W., Soininen H., Thein S., Shiovitz T., Pilcher G. (2012). Safety and tolerability of the γ-secretase inhibitor avagacestat in a phase 2 study of mild to moderate Alzheimer disease. Arch. Neurol..

[83] Crump C.J., Castro S.V., Wang F., Pozdnyakov N., Ballard T.E., Sisodia S.S., Bales K.R., Johnson D.S., Li Y.M. (2012). BMS-708,163 targets presenilin and lacks notch-sparing activity. Biochemistry.

[84] Ahn J.E., Carrieri C., Dela Cruz F., Fullerton T., Hajos-Korcsok E., He P., Kantaridis C., Leurent C., Liu R., Mancuso J. (2020). Pharmacokinetic and Pharmacodynamic Effects of a γ-Secretase Modulator, PF-06648671, on CSF Amyloid-β Peptides in Randomized Phase I Studies. Clin. Pharmacol. Ther..

[85] Ross J., Sharma S., Winston J., Nunez M., Bottini G., Franceschi M., Scarpini E., Frigerio E., Fiorentini F., Fernandez M. (2013). CHF5074 reduces biomarkers of neuroinflammation in patients with mild cognitive impairment: A 12-week, double-blind, placebo-controlled study. Curr. Alzheimer Res..

[86] Vellas B., Sol O., Snyder P.J., Ousset P.J., Haddad R., Maurin M., Lemarié J.C., Désiré L., Pando M.P. (2011). EHT0202 in Alzheimer’s disease: A 3-month, randomized, placebo-controlled, double-blind study. Curr. Alzheimer Res..

[87] Lee S.J., Nam E., Lee H.J., Savelieff M.G., Lim M.H. (2017). Towards an understanding of amyloid-β oligomers: Characterization, toxicity mechanisms, and inhibitors. Chem. Soc. Rev..

[88] Gervais F., Paquette J., Morissette C., Krzywkowski P., Yu M., Azzi M., Lacombe D., Kong X., Aman A., Laurin J. (2007). Targeting soluble Abeta peptide with Tramiprosate for the treatment of brain amyloidosis. Neurobiol. Aging.

[89] Gauthier S., Aisen P.S., Ferris S.H., Saumier D., Duong A., Haine D., Garceau D., Suhy J., Oh J., Lau W. (2009). Effect of tramiprosate in patients with mild-to-moderate Alzheimer’s disease: Exploratory analyses of the MRI sub-group of the Alphase study. J. Nutr. Health Aging.

[90] Hey J.A., Kocis P., Hort J., Abushakra S., Power A., Vyhnálek M., Yu J.Y., Tolar M. (2018). Discovery and Identification of an Endogenous Metabolite of Tramiprosate and Its Prodrug ALZ-801 that Inhibits Beta Amyloid Oligomer Formation in the Human Brain. CNS Drugs.

[91] Stark T., Lieblein T., Pohland M., Kalden E., Freund P., Zangl R., Grewal R., Heilemann M., Eckert G.P., Morgner N. (2017). Peptidomimetics That Inhibit and Partially Reverse the Aggregation of Aβ(1–42). Biochemistry.

[92] Pagano K., Tomaselli S., Molinari H., Ragona L. (2020). Natural Compounds as Inhibitors of Aβ Peptide Aggregation: Chemical Requirements and Molecular Mechanisms. Front. Neurosci..

[93] Du W.J., Guo J.J., Gao M.T., Hu S.Q., Dong X.Y., Han Y.F., Liu F.F., Jiang S., Sun Y. (2015). Brazilin inhibits amyloid β-protein fibrillogenesis, remodels amyloid fibrils and reduces amyloid cytotoxicity. Sci. Rep..

[94] Tang M., Wang Z., Zhou Y., Xu W., Li S., Wang L., Wei D., Qiao Z. (2013). A novel drug candidate for Alzheimer’s disease treatment: Gx-50 derived from Zanthoxylum bungeanum. J. Alzheimers Dis..

[95] Fu Z., Aucoin D., Ahmed M., Ziliox M., Van Nostrand W.E., Smith S.O. (2014). Capping of aβ42 oligomers by small molecule inhibitors. Biochemistry.

[96] Bieschke J., Russ J., Friedrich R.P., Ehrnhoefer D.E., Wobst H., Neugebauer K., Wanker E.E. (2010). EGCG remodels mature alpha-synuclein and amyloid-beta fibrils and reduces cellular toxicity. Proc. Natl. Acad. Sci. USA.

[97] Ahmed R., VanSchouwen B., Jafari N., Ni X., Ortega J., Melacini G. (2017). Molecular Mechanism for the (-)-Epigallocatechin Gallate-Induced Toxic to Nontoxic Remodeling of Aβ Oligomers. J. Am. Chem. Soc..

[98] Fan Q., Liu Y., Wang X., Zhang Z., Fu Y., Liu L., Wang P., Ma H., Ma H., Seeram N.P. (2020). Ginnalin A Inhibits Aggregation, Reverses Fibrillogenesis, and Alleviates Cytotoxicity of Amyloid β(1–42). ACS Chem. Neurosci..

[99] Lannfelt L., Blennow K., Zetterberg H., Batsman S., Ames D., Harrison J., Masters C.L., Targum S., Bush A.I., Murdoch R. (2008). Safety, efficacy, and biomarker findings of PBT2 in targeting Abeta as a modifying therapy for Alzheimer’s disease: A phase IIa, double-blind, randomised, placebo-controlled trial. Lancet Neurol..

[100] Faux N.G., Ritchie C.W., Gunn A., Rembach A., Tsatsanis A., Bedo J., Harrison J., Lannfelt L., Blennow K., Zetterberg H. (2010). PBT2 rapidly improves cognition in Alzheimer’s Disease: Additional phase II analyses. J. Alzheimers Dis..

[101] Turner R.S., Thomas R.G., Craft S., van Dyck C.H., Mintzer J., Reynolds B.A., Brewer J.B., Rissman R.A., Raman R., Aisen P.S. (2015). A randomized, double-blind, placebo-controlled trial of resveratrol for Alzheimer disease. Neurology.

[102] Moussa C., Hebron M., Huang X., Ahn J., Rissman R.A., Aisen P.S., Turner R.S. (2017). Resveratrol regulates neuro-inflammation and induces adaptive immunity in Alzheimer’s disease. J. Neuroinflamm..

[103] Kocis P., Tolar M., Yu J., Sinko W., Ray S., Blennow K., Fillit H., Hey J.A. (2017). Elucidating the Aβ42 Anti-Aggregation Mechanism of Action of Tramiprosate in Alzheimer’s Disease: Integrating Molecular Analytical Methods, Pharmacokinetic and Clinical Data. CNS Drugs.

[104] Schenk D., Barbour R., Dunn W., Gordon G., Grajeda H., Guido T., Hu K., Huang J., Johnson-Wood K., Khan K. (1999). Immunization with amyloid-beta attenuates Alzheimer-disease-like pathology in the PDAPP mouse. Nature.

[105] Pfeifer M., Boncristiano S., Bondolfi L., Stalder A., Deller T., Staufenbiel M., Mathews P.M., Jucker M. (2002). Cerebral hemorrhage after passive anti-Abeta immunotherapy. Science.

[106] Sevigny J., Chiao P., Bussière T., Weinreb P.H., Williams L., Maier M., Dunstan R., Salloway S., Chen T., Ling Y. (2016). The antibody aducanumab reduces Aβ plaques in Alzheimer’s disease. Nature.

[107] Avgerinos K.I., Ferrucci L., Kapogiannis D. (2021). Effects of monoclonal antibodies against amyloid-β on clinical and biomarker outcomes and adverse event risks: A systematic review and meta-analysis of phase III RCTs in Alzheimer’s disease. Ageing Res. Rev..

[108] Salloway S., Chalkias S., Barkhof F., Burkett P., Barakos J., Purcell D., Suhy J., Forrestal F., Tian Y., Umans K. (2022). Amyloid-Related Imaging Abnormalities in 2 Phase 3 Studies Evaluating Aducanumab in Patients with Early Alzheimer Disease. JAMA Neurol..

[109] Dhadda S., Kanekiyo M., Li D., Swanson C.J., Irizarry M., Berry S., Kramer L.D., Berry D.A. (2022). Consistency of efficacy results across various clinical measures and statistical methods in the lecanemab phase 2 trial of early Alzheimer’s disease. Alzheimers Res. Ther..

[110] McDade E., Cummings J.L., Dhadda S., Swanson C.J., Reyderman L., Kanekiyo M., Koyama A., Irizarry M., Kramer L.D., Bateman R.J. (2022). Lecanemab in patients with early Alzheimer’s disease: Detailed results on biomarker, cognitive, and clinical effects from the randomized and open-label extension of the phase 2 proof-of-concept study. Alzheimers Res. Ther..

[111] Ostrowitzki S., Lasser R.A., Dorflinger E., Scheltens P., Barkhof F., Nikolcheva T., Ashford E., Retout S., Hofmann C., Delmar P. (2017). A phase III randomized trial of gantenerumab in prodromal Alzheimer’s disease. Alzheimers Res. Ther..

[112] Salloway S., Farlow M., McDade E., Clifford D.B., Wang G., Llibre-Guerra J.J., Hitchcock J.M., Mills S.L., Santacruz A.M., Aschenbrenner A.J. (2021). A trial of gantenerumab or solanezumab in dominantly inherited Alzheimer’s disease. Nat. Med..

[113] Honig L.S., Vellas B., Woodward M., Boada M., Bullock R., Borrie M., Hager K., Andreasen N., Scarpini E., Liu-Seifert H. (2018). Trial of Solanezumab for Mild Dementia Due to Alzheimer’s Disease. N. Engl. J. Med..

[114] Ostrowitzki S., Bittner T., Sink K.M., Mackey H., Rabe C., Honig L.S., Cassetta E., Woodward M., Boada M., van Dyck C.H. (2022). Evaluating the Safety and Efficacy of Crenezumab vs Placebo in Adults with Early Alzheimer Disease: Two Phase 3 Randomized Placebo-Controlled Trials. JAMA Neurol..

[115] Adolfsson O., Pihlgren M., Toni N., Varisco Y., Buccarello A.L., Antoniello K., Lohmann S., Piorkowska K., Gafner V., Atwal J.K. (2012). An effector-reduced anti-β-amyloid (Aβ) antibody with unique aβ binding properties promotes neuroprotection and glial engulfment of Aβ. J. Neurosci..

[116] Lowe S.L., Willis B.A., Hawdon A., Natanegara F., Chua L., Foster J., Shcherbinin S., Ardayfio P., Sims J.R. (2021). Donanemab (LY3002813) dose-escalation study in Alzheimer’s disease. Alzheimer’s Dement. Transl. Res. Clin. Interv..

[117] Irizarry M.C., Sims J.R., Lowe S.L., Nakano M., Hawdon A., Willis B.A., Gonzales C., Liu P., Fujimoto S., Dean R.A. (2016). O4-08-06: Safety, Pharmacokinetics (PK), and Florbetapir F-18 Positron Emission Tomography (PET) After Multiple Dose Administration of LY3002813 Aβ-amyloid plaque-specific antibody, in Alzherimer’s Disease (AD). Alzheimer’s Dement..

[118] Lowe S.L., Duggan Evans C., Shcherbinin S., Cheng Y.J., Willis B.A., Gueorguieva I., Lo A.C., Fleisher A.S., Dage J.L., Ardayfio P. (2021). Donanemab (LY3002813) Phase 1b Study in Alzheimer’s Disease: Rapid and Sustained Reduction of Brain Amyloid Measured by Florbetapir F18 Imaging. J. Prev. Alzheimers Dis..

[119] Mintun M.A., Lo A.C., Duggan Evans C., Wessels A.M., Ardayfio P.A., Andersen S.W., Shcherbinin S., Sparks J., Sims J.R., Brys M. (2021). Donanemab in Early Alzheimer’s Disease. N. Engl. J. Med..

[120] Demattos R.B., Lu J., Tang Y., Racke M.M., Delong C.A., Tzaferis J.A., Hole J.T., Forster B.M., McDonnell P.C., Liu F. (2012). A plaque-specific antibody clears existing β-amyloid plaques in Alzheimer’s disease mice. Neuron.

[121] Lacosta A.M., Pascual-Lucas M., Pesini P., Casabona D., Pérez-Grijalba V., Marcos-Campos I., Sarasa L., Canudas J., Badi H., Monleón I. (2018). Safety, tolerability and immunogenicity of an active anti-Aβ(40) vaccine (ABvac40) in patients with Alzheimer’s disease: A randomised, double-blind, placebo-controlled, phase I trial. Alzheimers Res. Ther..

[122] Winblad B., Andreasen N., Minthon L., Floesser A., Imbert G., Dumortier T., Maguire R.P., Blennow K., Lundmark J., Staufenbiel M. (2012). Safety, tolerability, and antibody response of active Aβ immunotherapy with CAD106 in patients with Alzheimer’s disease: Randomised, double-blind, placebo-controlled, first-in-human study. Lancet Neurol..

[123] Wang C.Y., Wang P.N., Chiu M.J., Finstad C.L., Lin F., Lynn S., Tai Y.H., De Fang X., Zhao K., Hung C.H. (2017). UB-311, a novel UBITh(^®^) amyloid β peptide vaccine for mild Alzheimer’s disease. Alzheimer’s Dement. Transl. Res. Clin. Interv..

[124] Arndt J.W., Qian F., Smith B.A., Quan C., Kilambi K.P., Bush M.W., Walz T., Pepinsky R.B., Bussière T., Hamann S. (2018). Structural and kinetic basis for the selectivity of aducanumab for aggregated forms of amyloid-β. Sci. Rep..

[125] Tucker S., Möller C., Tegerstedt K., Lord A., Laudon H., Sjödahl J., Söderberg L., Spens E., Sahlin C., Waara E.R. (2015). The Murine Version of BAN2401 (mAb158) Selectively Reduces Amyloid-β Protofibrils in Brain and Cerebrospinal Fluid of tg-ArcSwe Mice. J. Alzheimer’s Dis..

[126] Söllvander S., Nikitidou E., Gallasch L., Zyśk M., Söderberg L., Sehlin D., Lannfelt L., Erlandsson A. (2018). The Aβ protofibril selective antibody mAb158 prevents accumulation of Aβ in astrocytes and rescues neurons from Aβ-induced cell death. J. Neuroinflamm..

[127] Dixit R., Ross J.L., Goldman Y.E., Holzbaur E.L. (2008). Differential regulation of dynein and kinesin motor proteins by tau. Science.

[128] Savastano A., Flores D., Kadavath H., Biernat J., Mandelkow E., Zweckstetter M. (2021). Disease-Associated Tau Phosphorylation Hinders Tubulin Assembly within Tau Condensates. Angew. Chem. Int. Ed. Engl..

[129] Liu F., Zaidi T., Iqbal K., Grundke-Iqbal I., Merkle R.K., Gong C.X. (2002). Role of glycosylation in hyperphosphorylation of tau in Alzheimer’s disease. FEBS Lett..

[130] Wilcock G.K., Gauthier S., Frisoni G.B., Jia J., Hardlund J.H., Moebius H.J., Bentham P., Kook K.A., Schelter B.O., Wischik D.J. (2018). Potential of Low Dose Leuco-Methylthioninium Bis(Hydromethanesulphonate) (LMTM) Monotherapy for Treatment of Mild Alzheimer’s Disease: Cohort Analysis as Modified Primary Outcome in a Phase III Clinical Trial. J. Alzheimers Dis..

[131] Tsai R.M., Miller Z., Koestler M., Rojas J.C., Ljubenkov P.A., Rosen H.J., Rabinovici G.D., Fagan A.M., Cobigo Y., Brown J.A. (2020). Reactions to Multiple Ascending Doses of the Microtubule Stabilizer TPI-287 in Patients With Alzheimer Disease, Progressive Supranuclear Palsy, and Corticobasal Syndrome: A Randomized Clinical Trial. JAMA Neurol..

[132] Novak P., Schmidt R., Kontsekova E., Zilka N., Kovacech B., Skrabana R., Vince-Kazmerova Z., Katina S., Fialova L., Prcina M. (2017). Safety and immunogenicity of the tau vaccine AADvac1 in patients with Alzheimer’s disease: A randomised, double-blind, placebo-controlled, phase 1 trial. Lancet Neurol..

[133] Novak P., Kovacech B., Katina S., Schmidt R., Scheltens P., Kontsekova E., Ropele S., Fialova L., Kramberger M., Paulenka-Ivanovova N. (2021). ADAMANT: A placebo-controlled randomized phase 2 study of AADvac1, an active immunotherapy against pathological tau in Alzheimer’s disease. Nature Aging.

[134] West T., Hu Y., Verghese P.B., Bateman R.J., Braunstein J.B., Fogelman I., Budur K., Florian H., Mendonca N., Holtzman D.M. (2017). Preclinical and Clinical Development of ABBV-8E12, a Humanized Anti-Tau Antibody, for Treatment of Alzheimer’s Disease and Other Tauopathies. J. Prev. Alzheimers Dis..

[135] Nobuhara C.K., DeVos S.L., Commins C., Wegmann S., Moore B.D., Roe A.D., Costantino I., Frosch M.P., Pitstick R., Carlson G.A. (2017). Tau Antibody Targeting Pathological Species Blocks Neuronal Uptake and Interneuron Propagation of Tau in Vitro. Am. J. Pathol..

[136] Courade J.P., Angers R., Mairet-Coello G., Pacico N., Tyson K., Lightwood D., Munro R., McMillan D., Griffin R., Baker T. (2018). Epitope determines efficacy of therapeutic anti-Tau antibodies in a functional assay with human Alzheimer Tau. Acta Neuropathol..

[137] Albert M., Mairet-Coello G., Danis C., Lieger S., Caillierez R., Carrier S., Skrobala E., Landrieu I., Michel A., Schmitt M. (2019). Prevention of tau seeding and propagation by immunotherapy with a central tau epitope antibody. Brain.

[138] Alam R., Driver D., Wu S., Lozano E., Key S.L., Hole J.T., Hayashi M.L., Lu J. (2017). Preclinical characterization of an antibody [LY3303560] targeting aggregated tau. Alzheimer’s Dement..

[139] Chai X., Wu S., Murray T.K., Kinley R., Cella C.V., Sims H., Buckner N., Hanmer J., Davies P., O’Neill M.J. (2011). Passive immunization with anti-Tau antibodies in two transgenic models: Reduction of Tau pathology and delay of disease progression. J. Biol. Chem..

[140] Kontsekova E., Zilka N., Kovacech B., Novak P., Novak M. (2014). First-in-man tau vaccine targeting structural determinants essential for pathological tau-tau interaction reduces tau oligomerisation and neurofibrillary degeneration in an Alzheimer’s disease model. Alzheimers Res. Ther..

[141] Theunis C., Crespo-Biel N., Gafner V., Pihlgren M., López-Deber M.P., Reis P., Hickman D.T., Adolfsson O., Chuard N., Ndao D.M. (2013). Efficacy and safety of a liposome-based vaccine against protein Tau, assessed in tau.P301L mice that model tauopathy. PLoS ONE.

[142] Guan P.P., Cao L.L., Wang P. (2021). Elevating the Levels of Calcium Ions Exacerbate Alzheimer’s Disease via Inducing the Production and Aggregation of β-Amyloid Protein and Phosphorylated Tau. Int. J. Mol. Sci..

[143] Mishra S.K., Hidau M., Rai S. (2021). Memantine and Ibuprofen pretreatment exerts anti-inflammatory effect against streptozotocin-induced astroglial inflammation via modulation of NMDA receptor-associated downstream calcium ion signaling. Inflammopharmacology.

[144] Chappell A.S., Gonzales C., Williams J., Witte M.M., Mohs R.C., Sperling R. (2007). AMPA potentiator treatment of cognitive deficits in Alzheimer disease. Neurology.

[145] Jhee S.S., Chappell A.S., Zarotsky V., Moran S.V., Rosenthal M., Kim E., Chalon S., Toublanc N., Brandt J., Coutant D.E. (2006). Multiple-dose plasma pharmacokinetic and safety study of LY450108 and LY451395 (AMPA receptor potentiators) and their concentration in cerebrospinal fluid in healthy human subjects. J. Clin. Pharmacol..

[146] Bernard K., Danober L., Thomas J.Y., Lebrun C., Muñoz C., Cordi A., Desos P., Lestage P., Morain P. (2010). DRUG FOCUS: S 18986: A positive allosteric modulator of AMPA-type glutamate receptors pharmacological profile of a novel cognitive enhancer. CNS Neurosci. Ther..

[147] Shevtsova E.F., Angelova P.R., Stelmashchuk O.A., Esteras N., Vasil’eva N.A., Maltsev A.V., Shevtsov P.N., Shaposhnikov A.V., Fisenko V.P., Bachurin S.O. (2022). Pharmacological sequestration of mitochondrial calcium uptake protects against dementia and β-amyloid neurotoxicity. Sci. Rep..

[148] Malek R., Maj M., Wnorowski A., Jóźwiak K., Martin H., Iriepa I., Moraleda I., Chabchoub F., Marco-Contelles J., Ismaili L. (2019). Multi-target 1,4-dihydropyridines showing calcium channel blockade and antioxidant capacity for Alzheimer’s disease therapy. Bioorg. Chem..

[149] Bhatt S., Puli L., Patil C.R. (2021). Role of reactive oxygen species in the progression of Alzheimer’s disease. Drug. Discov. Today.

[150] Balendra V., Singh S.K. (2021). Therapeutic potential of astaxanthin and superoxide dismutase in Alzheimer’s disease. Open. Biol..

[151] Ali T., Kim T., Rehman S.U., Khan M.S., Amin F.U., Khan M., Ikram M., Kim M.O. (2018). Natural Dietary Supplementation of Anthocyanins via PI3K/Akt/Nrf2/HO-1 Pathways Mitigate Oxidative Stress, Neurodegeneration, and Memory Impairment in a Mouse Model of Alzheimer’s Disease. Mol. Neurobiol..

[152] Wang C., Cai X., Hu W., Li Z., Kong F., Chen X., Wang D. (2019). Investigation of the neuroprotective effects of crocin via antioxidant activities in HT22 cells and in mice with Alzheimer’s disease. Int. J. Mol. Med..

[153] Yuan C., Shin M., Park Y., Choi B., Jang S., Lim C., Yun H.S., Lee I.S., Won S.Y., Cho K.S. (2021). Linalool Alleviates Aβ42-Induced Neurodegeneration via Suppressing ROS Production and Inflammation in Fly and Rat Models of Alzheimer’s Disease. Oxid. Med. Cell. Longev..

[154] Fei X., Zhang Y., Mei Y., Yue X., Jiang W., Ai L., Yu Y., Luo H., Li H., Luo W. (2021). Degradation of FA reduces Aβ neurotoxicity and Alzheimer-related phenotypes. Mol. Psychiatry.

[155] Letellier N., Gutierrez L.A., Pilorget C., Artaud F., Descatha A., Ozguler A., Goldberg M., Zins M., Elbaz A., Berr C. (2022). Association Between Occupational Exposure to Formaldehyde and Cognitive Impairment. Neurology.

[156] Li F., Yujie Q., Gong S., Zhang H., Ding S. (2020). Learning and memory impairment of mice caused by gaseous formaldehyde. Environ. Res..

[157] Liu X., Zhang Y., Wu R., Ye M., Zhao Y., Kang J., Ma P., Li J., Yang X. (2018). Acute formaldehyde exposure induced early Alzheimer-like changes in mouse brain. Toxicol. Mech. Methods.

[158] Zhai R., Rizak J., Zheng N., He X., Li Z., Yin Y., Su T., He Y., He R., Ma Y. (2018). Alzheimer’s Disease-Like Pathologies and Cognitive Impairments Induced by Formaldehyde in Non-Human Primates. Curr. Alzheimer Res..

[159] Tong Z., Han C., Qiang M., Wang W., Lv J., Zhang S., Luo W., Li H., Luo H., Zhou J. (2015). Age-related formaldehyde interferes with DNA methyltransferase function, causing memory loss in Alzheimer’s disease. Neurobiol. Aging.

[160] Kou Y., Zhao H., Cui D., Han H., Tong Z. (2022). Formaldehyde toxicity in age-related neurological dementia. Ageing Res. Rev..

[161] Tong Z., Zhang J., Luo W., Wang W., Li F., Li H., Luo H., Lu J., Zhou J., Wan Y. (2011). Urine formaldehyde level is inversely correlated to mini mental state examination scores in senile dementia. Neurobiol. Aging.

[162] Tong Z., Wang W., Luo W., Lv J., Li H., Luo H., Jia J., He R. (2017). Urine Formaldehyde Predicts Cognitive Impairment in Post-Stroke Dementia and Alzheimer’s Disease. J. Alzheimers Dis..

[163] Wang Y., Pan F., Xie F., He R., Guo Q. (2022). Correlation Between Urine Formaldehyde and Cognitive Abilities in the Clinical Spectrum of Alzheimer’s Disease. Front. Aging Neurosci..

[164] Boor P.J., Trent M.B., Lyles G.A., Tao M., Ansari G.A. (1992). Methylamine metabolism to formaldehyde by vascular semicarbazide-sensitive amine oxidase. Toxicology.

[165] Li Z.H., He X.P., Li H., He R.Q., Hu X.T. (2020). Age-associated changes in amyloid-β and formaldehyde concentrations in cerebrospinal fluid of rhesus monkeys. Zool. Res..

[166] Zhao Q., Lu J., Yao Z., Wang S., Zhu L., Wang J., Chen B. (2017). Upregulation of Aβ42 in the Brain and Bodily Fluids of Rhesus Monkeys with Aging. J. Mol. Neurosci..

[167] Tao R., Liao M., Wang Y., Wang H., Tan Y., Qin S., Wei W., Tang C., Liang X., Han Y. (2022). In Situ Imaging of Formaldehyde in Live Mice with High Spatiotemporal Resolution Reveals Aldehyde Dehydrogenase-2 as a Potential Target for Alzheimer’s Disease Treatment. Anal. Chem..

[168] Zhai R., Zheng N., Rizak J., Hu X. (2016). Evidence for Conversion of Methanol to Formaldehyde in Nonhuman Primate Brain. Anal. Cell. Pathol..

[169] Yang M., Miao J., Rizak J., Zhai R., Wang Z., Huma T., Li T., Zheng N., Wu S., Zheng Y. (2014). Alzheimer’s disease and methanol toxicity (part 2): Lessons from four rhesus macaques (Macaca mulatta) chronically fed methanol. J. Alzheimers Dis..

[170] Del Mar Hernandez M., Esteban M., Szabo P., Boada M., Unzeta M. (2005). Human plasma semicarbazide sensitive amine oxidase (SSAO), beta-amyloid protein and aging. Neurosci. Lett..

[171] Zhang J., Yue X., Luo H., Jiang W., Mei Y., Ai L., Gao G., Wu Y., Yang H., An J. (2019). Illumination with 630 nm Red Light Reduces Oxidative Stress and Restores Memory by Photo-Activating Catalase and Formaldehyde Dehydrogenase in SAMP8 Mice. Antioxid. Redox Signal..

[172] Tong Z., Han C., Luo W., Wang X., Li H., Luo H., Zhou J., Qi J., He R. (2013). Accumulated hippocampal formaldehyde induces age-dependent memory decline. Age.

[173] Lu J., Miao J., Su T., Liu Y., He R. (2013). Formaldehyde induces hyperphosphorylation and polymerization of Tau protein both in vitro and in vivo. Biochim. Biophys. Acta.

[174] Nie C.L., Wang X.S., Liu Y., Perrett S., He R.Q. (2007). Amyloid-like aggregates of neuronal tau induced by formaldehyde promote apoptosis of neuronal cells. BMC Neurosci..

[175] Yang X., Yang Y., Li G., Wang J., Yang E.S. (2008). Coenzyme Q10 attenuates beta-amyloid pathology in the aged transgenic mice with Alzheimer presenilin 1 mutation. J. Mol. Neurosci..

[176] Mei Y., Jiang C., Wan Y., Lv J., Jia J., Wang X., Yang X., Tong Z. (2015). Aging-associated formaldehyde-induced norepinephrine deficiency contributes to age-related memory decline. Aging Cell..

[177] He X., Li Z., Rizak J.D., Wu S., Wang Z., He R., Su M., Qin D., Wang J., Hu X. (2016). Resveratrol Attenuates Formaldehyde Induced Hyperphosphorylation of Tau Protein and Cytotoxicity in N2a Cells. Front. Neurosci..

[178] Corpas R., Griñán-Ferré C., Rodríguez-Farré E., Pallàs M., Sanfeliu C. (2019). Resveratrol Induces Brain Resilience Against Alzheimer Neurodegeneration Through Proteostasis Enhancement. Mol. Neurobiol..

[179] Jhaveri A., Deshpande P., Pattni B., Torchilin V. (2018). Transferrin-targeted, resveratrol-loaded liposomes for the treatment of glioblastoma. J. Control. Release.

[180] Sun J., Wei C., Liu Y., Xie W., Xu M., Zhou H., Liu J. (2019). Progressive release of mesoporous nano-selenium delivery system for the multi-channel synergistic treatment of Alzheimer’s disease. Biomaterials.

[181] Abozaid O.A.R., Sallam M.W., El-Sonbaty S., Aziza S., Emad B., Ahmed E.S.A. (2022). Resveratrol-Selenium Nanoparticles Alleviate Neuroinflammation and Neurotoxicity in a Rat Model of Alzheimer’s Disease by Regulating Sirt1/miRNA-134/GSK3β Expression. Biol. Trace Elem. Res..

[182] Takagaki A., Fukai K., Nanjo F., Hara Y. (2000). Reactivity of green tea catechins with formaldehyde. J. Wood Sci..

[183] Pervin M., Unno K., Takagaki A., Isemura M., Nakamura Y. (2019). Function of Green Tea Catechins in the Brain: Epigallocatechin Gallate and its Metabolites. Int. J. Mol. Sci..

[184] Huang J., Lu Y., Zhang B., Yang S., Zhang Q., Cui H., Lu X., Zhao Y., Yang X., Li R. (2019). Antagonistic effect of epigallocatechin-3-gallate on neurotoxicity induced by formaldehyde. Toxicology.

[185] Cano A., Ettcheto M., Chang J.H., Barroso E., Espina M., Kühne B.A., Barenys M., Auladell C., Folch J., Souto E.B. (2019). Dual-drug loaded nanoparticles of Epigallocatechin-3-gallate (EGCG)/Ascorbic acid enhance therapeutic efficacy of EGCG in a APPswe/PS1dE9 Alzheimer’s disease mice model. J. Control. Release.

[186] Rezai-Zadeh K., Arendash G.W., Hou H., Fernandez F., Jensen M., Runfeldt M., Shytle R.D., Tan J. (2008). Green tea epigallocatechin-3-gallate (EGCG) reduces beta-amyloid mediated cognitive impairment and modulates tau pathology in Alzheimer transgenic mice. Brain Res..

[187] Lee J.W., Lee Y.K., Ban J.O., Ha T.Y., Yun Y.P., Han S.B., Oh K.W., Hong J.T. (2009). Green tea (-)-epigallocatechin-3-gallate inhibits beta-amyloid-induced cognitive dysfunction through modification of secretase activity via inhibition of ERK and NF-kappaB pathways in mice. J. Nutr..

[188] Wang R. (2012). Physiological implications of hydrogen sulfide: A whiff exploration that blossomed. Physiol. Rev..

[189] Disbrow E., Stokes K.Y., Ledbetter C., Patterson J., Kelley R., Pardue S., Reekes T., Larmeu L., Batra V., Yuan S. (2021). Plasma hydrogen sulfide: A biomarker of Alzheimer’s disease and related dementias. Alzheimers Dement..

[190] Li X., Zhuang Y.Y., Wu L., Xie M., Gu H.F., Wang B., Tang X.Q. (2020). Hydrogen Sulfide Ameliorates Cognitive Dysfunction in Formaldehyde-Exposed Rats: Involvement in the Upregulation of Brain-Derived Neurotrophic Factor. Neuropsychobiology.

[191] Cao L., Cao X., Zhou Y., Nagpure B.V., Wu Z.Y., Hu L.F., Yang Y., Sethi G., Moore P.K., Bian J.S. (2018). Hydrogen sulfide inhibits ATP-induced neuroinflammation and Aβ(1–42) synthesis by suppressing the activation of STAT3 and cathepsin S. Brain Behav. Immun..

[192] Aboulhoda B.E., Rashed L.A., Ahmed H., Obaya E.M.M., Ibrahim W., Alkafass M.A.L., Abd El-Aal S.A., ShamsEldeen A.M. (2021). Hydrogen sulfide and mesenchymal stem cells-extracted microvesicles attenuate LPS-induced Alzheimer’s disease. J. Cell. Physiol..

[193] Xuan A., Long D., Li J., Ji W., Zhang M., Hong L., Liu J. (2012). Hydrogen sulfide attenuates spatial memory impairment and hippocampal neuroinflammation in β-amyloid rat model of Alzheimer’s disease. J. Neuroinflamm..

[194] Xi Y., Zhang Y., Zhou Y., Liu Q., Chen X., Liu X., Grune T., Shi L., Hou M., Liu Z. (2023). Effects of methionine intake on cognitive function in mild cognitive impairment patients and APP/PS1 Alzheimer’s Disease model mice: Role of the cystathionine-β-synthase/H(2)S pathway. Redox Biol..

[195] Stewart M.J., Malek K., Crabb D.W. (1996). Distribution of messenger RNAs for aldehyde dehydrogenase 1, aldehyde dehydrogenase 2, and aldehyde dehydrogenase 5 in human tissues. J. Investig. Med..

[196] Li R., Zhao Z., Sun M., Luo J., Xiao Y. (2016). ALDH2 gene polymorphism in different types of cancers and its clinical significance. Life Sci..

[197] Yang K., Ren J., Li X., Wang Z., Xue L., Cui S., Sang W., Xu T., Zhang J., Yu J. (2020). Prevention of aortic dissection and aneurysm via an ALDH2-mediated switch in vascular smooth muscle cell phenotype. Eur. Heart J..

[198] Fan Y., Chen Z., Ye T., Lin W., Wang Q., Lin B. (2018). Aldehyde dehydrogenase II rs671 polymorphism in essential hypertension. Clin. Chim. Acta.

[199] Tanaka F., Shiratori Y., Yokosuka O., Imazeki F., Tsukada Y., Omata M. (1996). High incidence of ADH2*1/ALDH2*1 genes among Japanese alcohol dependents and patients with alcoholic liver disease. Hepatology.

[200] Dingler F.A., Wang M., Mu A., Millington C.L., Oberbeck N., Watcham S., Pontel L.B., Kamimae-Lanning A.N., Langevin F., Nadler C. (2020). Two Aldehyde Clearance Systems Are Essential to Prevent Lethal Formaldehyde Accumulation in Mice and Humans. Mol. Cell..

[201] Chen C.H., Kraemer B.R., Mochly-Rosen D. (2022). ALDH2 variance in disease and populations. Dis. Model. Mech..

[202] Jin X., Long T., Chen H., Zeng Y., Zhang X., Yan L., Wu C. (2021). Associations of Alcohol Dehydrogenase and Aldehyde Dehydrogenase Polymorphism With Cognitive Impairment Among the Oldest-Old in China. Front. Aging Neurosci..

[203] Chen J., Huang W., Cheng C.H., Zhou L., Jiang G.B., Hu Y.Y. (2019). Association Between Aldehyde dehydrogenase-2 Polymorphisms and Risk of Alzheimer’s Disease and Parkinson’s Disease: A Meta-Analysis Based on 5,315 Individuals. Front. Neurol..

[204] Wang B., Wang J., Zhou S., Tan S., He X., Yang Z., Xie Y.C., Li S., Zheng C., Ma X. (2008). The association of mitochondrial aldehyde dehydrogenase gene (ALDH2) polymorphism with susceptibility to late-onset Alzheimer’s disease in Chinese. J. Neurol. Sci..

[205] Ohsawa I., Nishimaki K., Murakami Y., Suzuki Y., Ishikawa M., Ohta S. (2008). Age-dependent neurodegeneration accompanying memory loss in transgenic mice defective in mitochondrial aldehyde dehydrogenase 2 activity. J. Neurosci..

[206] D’Souza Y., Elharram A., Soon-Shiong R., Andrew R.D., Bennett B.M. (2015). Characterization of Aldh2 (-/-) mice as an age-related model of cognitive impairment and Alzheimer’s disease. Mol. Brain.

[207] Yang Y., Chen W., Wang X., Ge W. (2021). Impact of mitochondrial aldehyde dehydrogenase 2 on cognitive impairment in the AD model mouse. Acta Biochim. Biophys. Sin..

[208] Joshi A.U., Van Wassenhove L.D., Logas K.R., Minhas P.S., Andreasson K.I., Weinberg K.I., Chen C.H., Mochly-Rosen D. (2019). Aldehyde dehydrogenase 2 activity and aldehydic load contribute to neuroinflammation and Alzheimer’s disease related pathology. Acta Neuropathol. Commun..

[209] Abbott N.J., Patabendige A.A., Dolman D.E., Yusof S.R., Begley D.J. (2010). Structure and function of the blood-brain barrier. Neurobiol. Dis..

[210] Zenaro E., Piacentino G., Constantin G. (2017). The blood-brain barrier in Alzheimer’s disease. Neurobiol. Dis..

[211] Merlini M., Meyer E.P., Ulmann-Schuler A., Nitsch R.M. (2011). Vascular β-amyloid and early astrocyte alterations impair cerebrovascular function and cerebral metabolism in transgenic arcAβ mice. Acta Neuropathol..

[212] Ahn K.C., Learman C.R., Dunbar G.L., Maiti P., Jang W.C., Cha H.C., Song M.S. (2018). Characterization of Impaired Cerebrovascular Structure in APP/PS1 Mouse Brains. Neuroscience.

[213] Sengillo J.D., Winkler E.A., Walker C.T., Sullivan J.S., Johnson M., Zlokovic B.V. (2013). Deficiency in mural vascular cells coincides with blood-brain barrier disruption in Alzheimer’s disease. Brain Pathol..

[214] Sagare A.P., Bell R.D., Zhao Z., Ma Q., Winkler E.A., Ramanathan A., Zlokovic B.V. (2013). Pericyte loss influences Alzheimer-like neurodegeneration in mice. Nat. Commun..

[215] Donahue J.E., Flaherty S.L., Johanson C.E., Duncan J.A., Silverberg G.D., Miller M.C., Tavares R., Yang W., Wu Q., Sabo E. (2006). RAGE, LRP-1, and amyloid-beta protein in Alzheimer’s disease. Acta Neuropathol..

[216] He X., Wang X., Yang L., Yang Z., Yu W., Wang Y., Liu R., Chen M., Gao H. (2022). Intelligent lesion blood-brain barrier targeting nano-missiles for Alzheimer’s disease treatment by anti-neuroinflammation and neuroprotection. Acta Pharm. Sin. B.

[217] Han H., Li K., Yan J., Zhu K., Fu Y. (2012). An in vivo study with an MRI tracer method reveals the biophysical properties of interstitial fluid in the rat brain. Sci. China Life Sci..

[218] Syková E., Nicholson C. (2008). Diffusion in brain extracellular space. Physiol. Rev..

[219] Shoji M., Golde T.E., Ghiso J., Cheung T.T., Estus S., Shaffer L.M., Cai X.D., McKay D.M., Tintner R., Frangione B. (1992). Production of the Alzheimer amyloid beta protein by normal proteolytic processing. Science.

[220] Syková E., Vorísek I., Antonova T., Mazel T., Meyer-Luehmann M., Jucker M., Hájek M., Ort M., Bures J. (2005). Changes in extracellular space size and geometry in APP23 transgenic mice: A model of Alzheimer’s disease. Proc. Natl. Acad. Sci. USA.

[221] Mueggler T., Meyer-Luehmann M., Rausch M., Staufenbiel M., Jucker M., Rudin M. (2004). Restricted diffusion in the brain of transgenic mice with cerebral amyloidosis. Eur. J. Neurosci..

[222] Xu F., Hongbin H., Yan J., Chen H., He Q., Xu W., Zhu N., Zhang H., Zhou F., Lee K. (2011). Greatly improved neuroprotective efficiency of citicoline by stereotactic delivery in treatment of ischemic injury. Drug. Deliv..

[223] Kong S.D., Lee J., Ramachandran S., Eliceiri B.P., Shubayev V.I., Lal R., Jin S. (2012). Magnetic targeting of nanoparticles across the intact blood-brain barrier. J. Control. Release.

[224] Gkountas A.A., Polychronopoulos N.D., Sofiadis G.N., Karvelas E.G., Spyrou L.A., Sarris I.E. (2021). Simulation of magnetic nanoparticles crossing through a simplified blood-brain barrier model for Glioblastoma multiforme treatment. Comput. Methods Programs Biomed..

[225] Laurent S., Saei A.A., Behzadi S., Panahifar A., Mahmoudi M. (2014). Superparamagnetic iron oxide nanoparticles for delivery of therapeutic agents: Opportunities and challenges. Expert. Opin. Drug. Deliv..

[226] Pedram M.Z., Shamloo A., Alasty A., Ghafar-Zadeh E. (2016). Optimal Magnetic Field for Crossing Super-Para-Magnetic Nanoparticles through the Brain Blood Barrier: A Computational Approach. Biosensors.

[227] Huang Y., Zhang B., Xie S., Yang B., Xu Q., Tan J. (2016). Superparamagnetic Iron Oxide Nanoparticles Modified with Tween 80 Pass through the Intact Blood-Brain Barrier in Rats under Magnetic Field. ACS Appl. Mater. Interfaces.

[228] Chen J., Yuan M., Madison C.A., Eitan S., Wang Y. (2022). Blood-brain barrier crossing using magnetic stimulated nanoparticles. J. Control. Release.

[229] Hong K.S., Khan M.N.A., Ghafoor U. (2022). Non-invasive transcranial electrical brain stimulation guided by functional near-infrared spectroscopy for targeted neuromodulation: A review. J. Neural Eng..

[230] Cai Y., Wei Z., Song C., Tang C., Han W., Dong X. (2019). Optical nano-agents in the second near-infrared window for biomedical applications. Chem. Soc. Rev..

[231] Zheng Y., Zhang Z., Liu Q., Wang Y., Hao J., Kang Z., Wang C., Zhao X., Liu Y., Shi L. (2021). A near-infrared light-excitable immunomodulating nano-photosensitizer for effective photoimmunotherapy. Biomater. Sci..

[232] Wang J., Gu Y., Liu X., Fan Y., Zhang Y., Yi C., Cheng C., Yang M. (2022). Near-Infrared Photothermally Enhanced Photo-Oxygenation for Inhibition of Amyloid-β Aggregation Based on RVG-Conjugated Porphyrinic Metal-Organic Framework and Indocyanine Green Nanoplatform. Int. J. Mol. Sci..

[233] Liu W., Zhang H., Dong X., Sun Y. (2022). Composite of gold nanoclusters and basified human serum albumin significantly boosts the inhibition of Alzheimer’s β-amyloid by photo-oxygenation. Acta Biomater..

[234] Rezai A.R., Ranjan M., D’Haese P.F., Haut M.W., Carpenter J., Najib U., Mehta R.I., Chazen J.L., Zibly Z., Yates J.R. (2020). Noninvasive hippocampal blood-brain barrier opening in Alzheimer’s disease with focused ultrasound. Proc. Natl. Acad. Sci. USA.

[235] Mehta R.I., Carpenter J.S., Mehta R.I., Haut M.W., Ranjan M., Najib U., Lockman P., Wang P., D’Haese P.F., Rezai A.R. (2021). Blood-Brain Barrier Opening with MRI-guided Focused Ultrasound Elicits Meningeal Venous Permeability in Humans with Early Alzheimer Disease. Radiology.

[236] Rezai A.R., Ranjan M., Haut M.W., Carpenter J., D’Haese P.F., Mehta R.I., Najib U., Wang P., Claassen D.O., Chazen J.L. (2022). Focused ultrasound-mediated blood-brain barrier opening in Alzheimer’s disease: Long-term safety, imaging, and cognitive outcomes. J. Neurosurg..

[237] Wasielewska J.M., Chaves J.C.S., Johnston R.L., Milton L.A., Hernández D., Chen L., Song J., Lee W., Leinenga G., Nisbet R.M. (2022). A sporadic Alzheimer’s blood-brain barrier model for developing ultrasound-mediated delivery of Aducanumab and anti-Tau antibodies. Theranostics.

[238] Rich M.C., Sherwood J., Bartley A.F., Whitsitt Q.A., Lee M., Willoughby W.R., Dobrunz L.E., Bao Y., Lubin F.D., Bolding M. (2020). Focused ultrasound blood brain barrier opening mediated delivery of MRI-visible albumin nanoclusters to the rat brain for localized drug delivery with temporal control. J. Control. Release.

[239] Liu Y., Gong Y., Xie W., Huang A., Yuan X., Zhou H., Zhu X., Chen X., Liu J., Liu J. (2020). Microbubbles in combination with focused ultrasound for the delivery of quercetin-modified sulfur nanoparticles through the blood brain barrier into the brain parenchyma and relief of endoplasmic reticulum stress to treat Alzheimer’s disease. Nanoscale.

[240] Ramos-Zaldívar H.M., Polakovicova I., Salas-Huenuleo E., Corvalán A.H., Kogan M.J., Yefi C.P., Andia M.E. (2022). Extracellular vesicles through the blood-brain barrier: A review. Fluids Barriers CNS.

[241] Morad G., Carman C.V., Hagedorn E.J., Perlin J.R., Zon L.I., Mustafaoglu N., Park T.E., Ingber D.E., Daisy C.C., Moses M.A. (2019). Tumor-Derived Extracellular Vesicles Breach the Intact Blood-Brain Barrier via Transcytosis. ACS Nano.

[242] Cui G.H., Guo H.D., Li H., Zhai Y., Gong Z.B., Wu J., Liu J.S., Dong Y.R., Hou S.X., Liu J.R. (2019). RVG-modified exosomes derived from mesenchymal stem cells rescue memory deficits by regulating inflammatory responses in a mouse model of Alzheimer’s disease. Immun. Ageing.

[243] Zhang X., He T., Chai Z., Samulski R.J., Li C. (2018). Blood-brain barrier shuttle peptides enhance AAV transduction in the brain after systemic administration. Biomaterials.

[244] Zhang X., Chai Z., Lee Dobbins A., Itano M.S., Askew C., Miao Z., Niu H., Samulski R.J., Li C. (2022). Customized blood-brain barrier shuttle peptide to increase AAV9 vector crossing the BBB and augment transduction in the brain. Biomaterials.

[245] Hersh D.S., Anastasiadis P., Mohammadabadi A., Nguyen B.A., Guo S., Winkles J.A., Kim A.J., Gullapalli R., Keller A., Frenkel V. (2018). MR-guided transcranial focused ultrasound safely enhances interstitial dispersion of large polymeric nanoparticles in the living brain. PLoS ONE.

